# Complementary LC-MS/MS-Based *N*-Glycan, *N*-Glycopeptide, and Intact *N*-Glycoprotein Profiling Reveals Unconventional Asn71-Glycosylation of Human Neutrophil Cathepsin G

**DOI:** 10.3390/biom5031832

**Published:** 2015-08-12

**Authors:** Ian Loke, Nicolle H. Packer, Morten Thaysen-Andersen

**Affiliations:** Department of Chemistry and Biomolecular Sciences, Macquarie University, North Ryde, Sydney 2109, Australia; E-Mails: ian.loke@students.mq.edu.au (I.L.); nicki.packer@mq.edu.au (N.H.P.)

**Keywords:** neutrophil, cathepsin G, *N*-glycan, glycopeptide, glycoprotein, chitobiose, paucimannose, *N*-acetylglucosamine, glycomics, azurophilic granule

## Abstract

Neutrophil cathepsin G (nCG) is a central serine protease in the human innate immune system, but the importance of its *N*-glycosylation remains largely undescribed. To facilitate such investigations, we here use complementary LC-MS/MS-based *N*-glycan, *N*-glycopeptide, and intact glycoprotein profiling to accurately establish the micro- and macro-heterogeneity of nCG from healthy individuals. The fully occupied Asn71 carried unconventional *N*-glycosylation consisting of truncated chitobiose core (GlcNAcβ: 55.2%; Fucα1,6GlcNAcβ: 22.7%), paucimannosidic *N*-glycans (Manβ1,4GlcNAcβ1,4GlcNAcβ: 10.6%; Manβ1,4GlcNAcβ1,4(Fucα1,6)GlcNAcβ: 7.9%; Manα1,6Manβ1,4GlcNAcβ1,4GlcNAcβ: 3.7%, trace level of Manα1,6Manβ1,4GlcNAcβ1,4(Fucα1,6)GlcNAcβ), and trace levels of monoantennary α2,6- and α2,3-sialylated complex *N*-glycans. High-resolution/mass accuracy LC-MS profiling of intact nCG confirmed the Asn71-glycoprofile and identified two C-terminal truncation variants at Arg243 (57.8%) and Ser244 (42.2%), both displaying oxidation of solvent-accessible Met152. Asn71 appeared proximal (~19 Å) to the active site of nCG, but due to the truncated nature of Asn71-glycans (~5–17 Å) we questioned their direct modulation of the proteolytic activity of the protein. This work highlights the continued requirement of using complementary technologies to accurately profile even relatively simple glycoproteins and illustrates important challenges associated with the analysis of unconventional protein N-glycosylation. Importantly, this study now facilitates investigation of the functional role of nCG Asn71-glycosylation.

## 1. Introduction

Neutrophil cathepsin G (nCG) is an important serine protease produced and stored predominately in the azurophilic granules of resting human neutrophils [1]. Facilitated by its proteolytic and anti-microbial activities [2], nCG is a key player in multiple physiological and pathophysiological processes e.g., innate immune defense against pathogens [3], vascular homeostasis [4], and inflammatory response [5].

**Figure 1:**
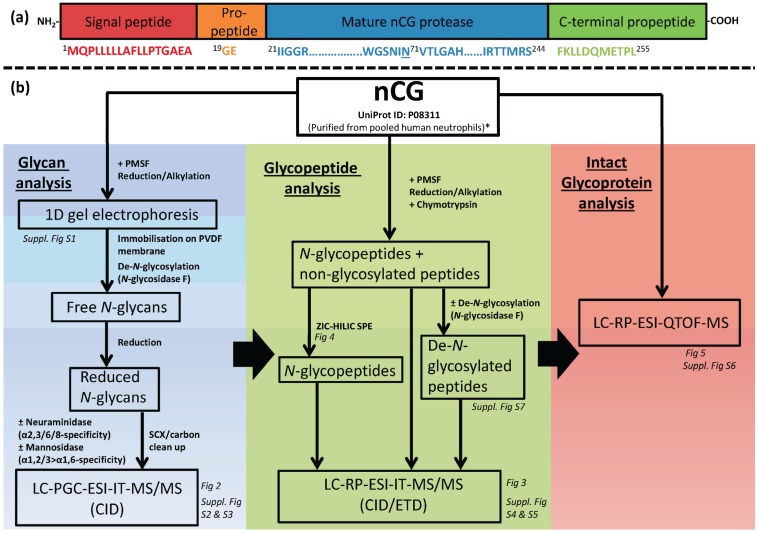
(**a**) Diagram showing the protein sequence of full length nCG consisting of a signal peptide, propeptide, the protease sequence, and a C-terminal propeptide. Numbering is based on the unprocessed nCG polypeptide chain; (**b**) Experimental workflow outlining the characterization of nCG *N*-glycoprofiling. The LC-MS/MS-centric experiments were divided into the three investigated analyte levels *i.e.*, *N*-glycans, *N*-glycopeptides, and intact glycoprotein. The relevant figures presenting the structural information extracted from the different analyses are indicated. * Other interfering human neutrophil glycoproteins of significant abundance were detected in the investigated protein preparation *i.e.*, azurocidin and neutrophil elastase (NE).

The nascent polypeptide chain of nCG (255 amino acid residues, ~28 kDa) is matured partly by the removal of an N-terminal signal- and pro-peptide and by a C-terminal cleavage at Ser244-Phe245 (unprocessed nCG polypeptide chain numbering hereafter) (Figure 1a) [6,7]. The crystal structure of nCG shows the catalytic triad characteristic of serine proteases forming the active site of the protein at His64, Asp108, and Ser201 [8]. The protein structure, catalytic activity, and function of nCG as a serine protease in the human immune system have been thoroughly reviewed [9,10]. It was established that nCG forms three intra-molecular disulfide bonds and presents a single potential asparagine (*N*)-linked glycosylation site at Asn71 [11].

Although the *N*-glycosylation of nCG has been studied previously, the micro- and macro-heterogeneity of nCG remain incompletely understood. Utilizing nuclear magnetic resonance, the presence of a biantennary disialylated complex and paucimannosidic *N*-glycans have been documented [12]. However, the distribution of the Asn71-glycoforms, glycosylation site occupancy, and presence of other post-translational modifications (PTMs) on nCG were not described. Knowledge of any potential involvement of Asn71-glycosylation in the function of nCG is similarly sparse: to our knowledge there is only a single *in vitro* study using a mutated nCG variant lacking the Asn71-glycosylation site, indicating that glycosylation at this site is not essential for the biosynthesis, stability, post-translational enzymatic activation, and granule sorting of nCG [13]. Nonetheless, Asn71-glycosylation may still play an important role in the function of nCG. In order to understand the influence of *N*-glycosylation on protein function, it is essential to accurately map the glycan structures and their distribution in a site-specific manner.

We recently identified the presence of an under-reported class of truncated human *N*-glycoproteins in neutrophil-rich and bacteria-infected sputum [14,15]. This so-called paucimannosylation of proteins is defined by the presence of a complete or partial trimannosyl-chitobiose *N-*linked core with the general monosaccharide composition *N*-acetylglucosamine(GlcNAc)_2_mannose(Man)_1-3_fucose(Fuc)_0-1_. Notably, these unusual glycoepitopes were found to be carried in abundance by proteins localizing to the azurophilic granules of neutrophils, including the bioactive myeloperoxidase, azurocidin, and neutrophil elastase (NE). We suggested that paucimannosylation may play crucial modulatory roles in the human innate immune system. Thus, detailing the exact *N*-glycosylation of nCG, which is also a known azurophilic granule protein [16], will facilitate further insights into the function of the unusual compartment-specific *N*-glycosylation of neutrophil proteins.

To this end, we here use complementary liquid chromatography-tandem mass spectrometry (LC-MS/MS) technologies available in modern glycoscience to show that nCG carries unexpected Asn71-glycosylation, including truncated chitobiose cores, and paucimannosidic and monoantennary monosialylated complex glycans. Documenting the Asn71*-*glycosylation and its relationship to other PTMs of the polypeptide chain of nCG advances our understanding of its molecular heterogeneity, and will enable more exact structure-function studies. Importantly, this study demonstrates the continued requirement for utilizing an array of analytical technologies to perform accurate and confident structural characterization of human *N*-glycoproteins carrying unconventional carbohydrates.

## 2. Results and Discussion

### 2.1. Design of Study—Three Analysis Levels were Used to Complete the nCG Glycoprofiling

Multiple LC-MS/MS-based approaches were utilized to achieve a deep site-specific structural characterization of nCG *N*-glycosylation (Figure 1b). These complementary techniques comprised the analysis of *N*-glycosidase F-released *N*-glycans, chymotrypsin-generated *N*-glycopeptides, and intact nCG. Porous graphitized carbon (PGC) LC electrospray ionization (ESI) resonance activation collision induced dissociated (CID) MS/MS is a well-documented technology to characterize reduced, underivatized *N*-glycans released from glycoproteins [17,18,19,20,21,22]. Such information-rich analysis provides the detailed structure of the individual *N*-glycans, their isomers, and their relative distribution, albeit in a protein and site-unspecific manner. The reduced *N*-glycan alditols released from nCG were analyzed in negative ion polarity, allowing for simultaneous detection of neutral and acidic glycans [23]. Parallel treatments with multiple exoglycosidases *i.e.*, α2,3/6/8-linkage unspecific and α2,3-linkage-specific sialidases and (α1,2/3 > α1,6) mannosidase were used together with the PGC-LC-MS/MS glycan profiling retention data and the established pathway knowledge of human *N*-glycosylation [14,24] to define the indicated terminal monosaccharide residues and their glycosidic linkages.

The site-specific *N*-glycosylation analysis was carried out on a reversed phase (RP) LC-MS/MS ion trap platform by identifying Asn71-glycopeptides. nCG was treated with phenylmethylsulfonyl fluoride (PMSF) to prevent autoproteolysis (Figure S1). Asn71-glycopeptides falling into an MS-friendly mass range (<4 kDa) were achieved using chymotrypsin digestion rather than using conventional trypsin digestion. Orthogonal CID and electron transfer dissociation (ETD) glycopeptide fragmentation, as previously described [25,26,27,28], facilitated knowledge of the glycosylation site, occupancy level, and site-specific distribution of Asn71-glycans. Zwitterionic hydrophilic interaction liquid chromatography (ZIC-HILIC)-based solid phase extraction (SPE) was employed to enrich for, and enhance the stoichiometry of, nCG *N*-glycopeptides, and to reduce the suppression effect of non-glycosylated peptides [29,30,31]. Although exoglycosidase treatment may be performed on the glycopeptide level to confirm the site-specific glycan linkages [32], this was not performed in this study.

Finally, intact nCG glycoprotein was profiled using high-resolution/high mass accuracy quadrupole time-of-flight (QTOF) LC-ESI-MS, generating a more holistic qualitative and quantitative picture of the connectivity of all PTMs displayed by the nCG polypeptide chain [33].

### 2.2. N-Glycome Profiling Indicates Unconventional nCG N-Glycosylation

**Figure 2:**
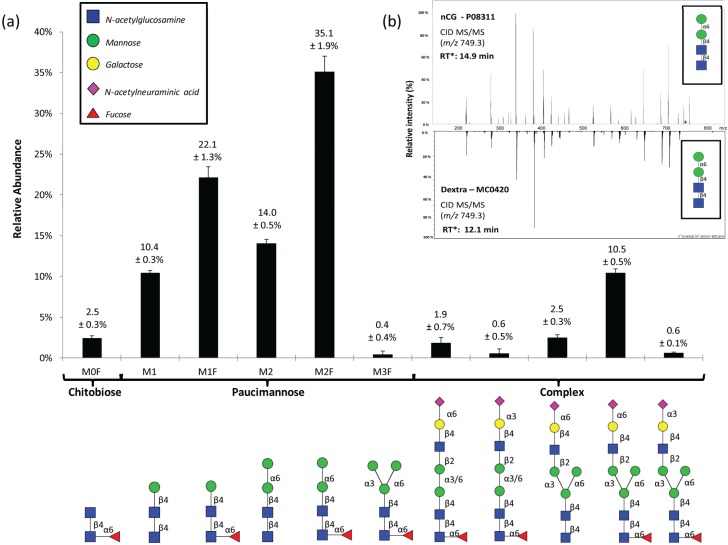
(**a**) The PGC-LC-MS/MS-based *N*-glycome profiling revealed 11 *N*-glycans comprising chitobiose core, paucimannosidic, and complex type *N*-glycans. The structures, linkages, and relative abundances of the individual *N*-glycans are illustrated. Data points are plotted as mean ± SD, *n* = 3. Symbols are used according to the Consortium for Functional Glycomics / Essentials of Glycobiology notation (see insert). All *N*-glycans were observed in their reduced (alditol) form; (**b**) CID-MS/MS and PGC-LC retention time (* calculated relative to M3F in each profile) matching of the paucimannosidic M2 to an identical glycan reference compound was performed. See Figure S3 for *de novo* CID-MS/MS characterization of all observed *N*-glycans analyzed under negative ionization polarity.

The *N*-glycome profiling was performed on PMSF-treated nCG isolated to single gel band purity as assessed by protein staining to minimize the risk of *N*-glycan contributions from interfering glycoproteins (Figure S1). However, as later demonstrated, the single gel band of the commercially prepared nCG turned out to contain multiple contaminating glycoproteins, illustrating that the risk of contamination may be reduced by SDS-PAGE separation, but cannot be eliminated completely. PGC-LC-MS/MS of *N*-glycans released from this gel band revealed an abundance of five paucimannosidic *N*-glycans (individual structures hereafter called M1, M1F, M2, M2F, and M3F, where M denotes the mannosylated chitobiose core (GlcNAcβ1,4GlcNAcβ) and F denotes α1,6-core fucosylation) and lower levels of other structures *i.e.*, a further truncated amannosylated chitobiose core type *N*-glycan (hereafter called M0F) and monoantennary complex sialo-*N*-glycans (Figure 2a). The specific linkages and branching configurations of the *N*-glycans were deduced from their monoisotopic masses, relative PGC-LC retention times, presence of diagnostic and other B-/C-/Y-/Z- and cross ring fragment ions (Figures S2 and S3 and Table S1) as previously described [21,34,35,36], and their response to specific exoglycosidase digestions [35]. *De novo* resonance activation (ion trap) CID-MS/MS sequencing, as well as the matching of ion trap CID-MS/MS spectra and the relative PGC-LC retention time to glycan reference compounds, were also performed to confirm the mannose arm linkage configurations of the paucimannosidic structures (Figure 2b).

The M2F α1,6-isomer of nCG was the most abundant *N*-glycan, in agreement with our recent *N*-glycome profiles of isolated human neutrophils and neutrophil-rich pathogen-infected sputum [14,15]. The corresponding α1,3-isomers of M2 and M2F were absent, in line with previous observations made in azurophilic granule proteins derived from human neutrophils [15,24] and the known preferential hydrolysis of α1,3-linked mannose of M3(F) by human α-D-mannosidase in the biosynthetic machinery [37]. Protein paucimannosylation has recently been indicated to exist in human cells and tissues other than neutrophils e.g., in inflammation and cancer [38,39,40]. Due to their abundance and presence on intact proteins in human neutrophils [24,41,42], we recently suggested that paucimannosylation is not simply a degradation product but should be considered as a separate non-conventional type of expressed human *N*-glycoproteins in addition to the more conventional high mannose, hybrid, and complex type glycoproteins [15].

Interestingly, the monoantennary complex *N*-glycans carrying an intact core-fucosylated trimannosyl-chitobiose core were found to display both α2,6- and α2,3-linked sialylation on the 3'-mannose arm, while the single non-core fucosylated monoantennary complex *N*-glycan was exclusively α2,6-sialylated. The less abundant related mannose-truncated (bimannosyl-chitobiose core) complex *N*-glycans carried both α2,6- and α2,3-linked sialylation on the 3'- or 6'-mannose arm, in agreement with previous studies reporting the presence of these unconventional monoantennary complex *N*-glycans on neutrophil proteins [24,43]. These structures did not contain the intact common trimannosyl-chitobiose cores typically found in mammalian *N*-glycans, illustrating that it may be relevant to include such unconventional structures (or the corresponding monosaccharide compositions) when using search engines (e.g., Byonic) for glycomics/glycoproteomics experiments. Upon consultation of an established glycan structure database (*i.e.*, UniCarbKB), we noticed that monoantennary core-truncated *N*-glycans have not yet been deposited. We ensured that artificial generation of these truncated structures by any contaminating glycosidase activity during the *N*-glycosidase F based *N*-glycan release was ruled out by performing parallel treatment and analysis of a number of model glycoproteins (*i.e.*, fetuin, ovalbumin, and RNase B) subjected to identical conditions as for nCG. However, further characterization is still needed to confirm the structure, function, and (sub)cellular origin of these unconventional species.

### 2.3. Site-Specific Asn71-Glycopeptide Profiling Uncovers Single GlcNAcβ and Fucα1,6GlcNAcβ on nCG and Reveals Significant Presence of Other Interfering N-Glycoproteins

**Figure 3:**
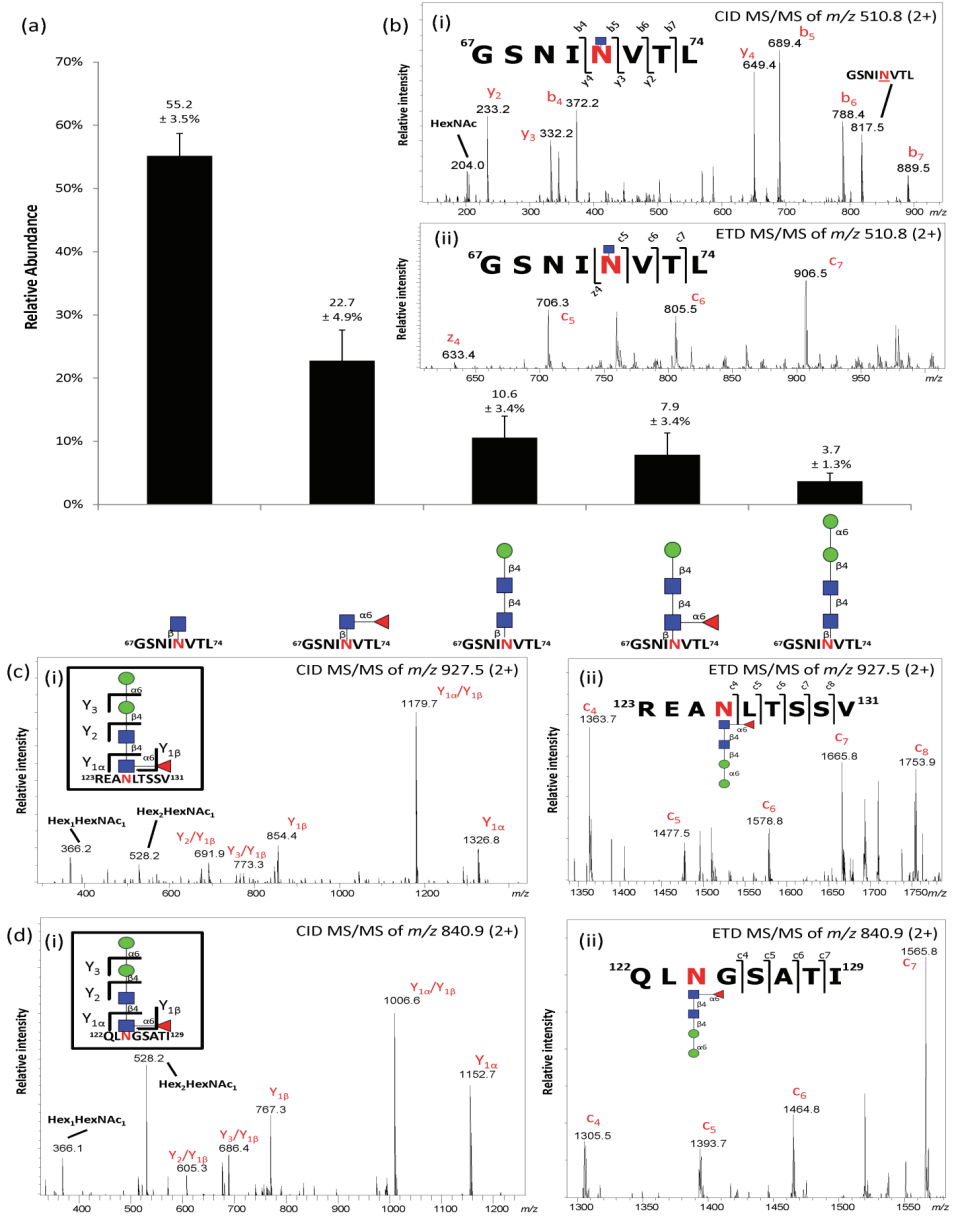
(**a**) Profiling of unenriched chymotryptic Asn71-glycopeptides derived from nCG in positive ionization polarity. Data points are plotted as mean ± SD, *n* = 3; (**b**) (i) CID-and (ii) ETD-MS/MS of the predominant chymotryptic GlcNAcβAsn71-glycopeptide variant (GSNI**N**VTL); (**c**,**d**) (i) CID- and (ii) ETD-MS/MS of the chymotryptic M2F-containing *N*-glycopeptide originating from the interfering neutrophil proteins *i.e.*, (**c**) azurocidin and (**d**) NE covering the *N*-glycosylation sites Asn126 and Asn124, respectively. See Figure S4 for glycopeptides covering other observed sites of azurocidin and NE. See also Table 1 for summary of all observed glycoforms of the three *N*-glycoproteins identified in the protein preparation.

RP-LC-CID/ETD MS/MS of the unenriched chymotryptic peptide mixture of nCG facilitated the identification of five abundant glycoforms of the Asn71-glycopeptide GSNI**N**VTL, including the attachment of single GlcNAcβ (55.2% ± 3.5%) and Fucα1,6GlcNAcβ (22.7 ± 4.9%) structures (Figure 3a,b). Other lower abundant chymotryptic Asn71-glycopeptide variants were also observed carrying the same set of *N*-glycans *i.e.*, the GSNI**N**V peptide (see Figure S4 and Table S2). Surprisingly, these abundant Asn71-glycoforms did not reflect the *N*-glycome relative distribution, probably due to the lack of PGC-LC retention of mono- and di-saccharides [20] and the resistance to digestion by *N*-glycosidase F of the truncated chitobiose core type structures [44]. GlcNAcβAsn- and Fucα1,6GlcNAcβAsn-glycosylation has been suggested to be present in the mouse synaptosomes and liver [45,46], as well as in mammalian cell lines [47] and invertebrates [48,49], but remains an under-reported and under-investigated class of *N*-glycans as they have previously been assumed to be non-functional degradation products.

**Table 1:** Semi-quantitative overview of the site-specific *N*-glycosylation of human nCG, azurocidin and NE observed in this study. “xxxx” denotes glycoforms of high abundance, “xxx” glycoforms of intermediate abundance, “xx” glycoforms of low abundance and “x” trace (non-quantifiable) glycoform abundance.

*N*-glycan Structure	nCG	Azurocidin		NE	
	Asn71	Asn126	Asn171	Asn124	Asn173
GlcNAcβ	xxxx				
Fucα1,6GlcNAcβ	xxxx				
Manβ1,4GlcNAcβ1,4GlcNAcβ (M1)	xxx				
Manβ1,4GlcNAcβ1,4(Fucα1,6) GlcNAcβ (M1F)	xxx		xx		
Manα1,6Manβ1,4GlcNAcβ1,4 GlcNAcβ (M2)	xxx				
Manα1,6Manβ1,4GlcNAcβ1,4 (Fucα1,6)GlcNAcβ (M2F)	x	xxxx	xxxx	xxxx	xxxx
Trimannosyl-chitobiose core monoantennary core fucosylated α2,6-monosialylated	x				
Trimannosyl-chitobiose core monoantennary core fucosylated α2,3-monosialylated	x				
Trimannosyl-chitobiose core monoantennary α2,6-monosialylated	x				
Bimannosyl-chitobiose core monoantennary core fucosylated α2,6-monosialylated	x				
Bimannosyl-chitobiose core monoantennary core fucosylated α2,3-monosialylated	x				

Importantly, paucimannosylation (M1, M1F, M2) corresponded to only ~20% of the total Asn71-glycopeptides derived from nCG. The glycopeptide profiling also indicated that nCG carried no or a negligible amount of M0F, M2F, and complex structures, all of which appeared at relatively high abundance in the released *N*-glycome. Comparable ionization response factors were estimated for the population of the glycopeptide species carrying the neutral chitobiose core and paucimannose glycans when analyzed in positive polarity MS [30]. This was supported by their similar LC elution window *i.e.*, 39 ± 1 min, giving them approximately equal solvent conditions for the ionization. However, the sialylated monoantennary complex *N*-glycopeptides were likely under-represented in the positive ion polarity used for the glycopeptide analysis relative to the neutral glycopeptides carrying the paucimannose, chitobiose core, and single GlcNAcβ variants [30], possibly also creating a slight bias towards the sialoglycans in the glycomic profile obtained in negative ion polarity mode. This discrepancy was also explained by the identification of M1F and, in particular, M2F glycopeptides on two *N*-glycosylation sites of azurocidin (Asn126 and Asn171) and NE (Asn124 and Asn173) (Figure 3c–d). Hence, it became evident that nCG was not isolated to absolute purity and that the interfering azurocidin and NE had dramatically skewed the *N*-glycomic profile towards a more paucimannose-rich profile. The presence of nCG, azurocidin, and NE observed may relate to their similar nature and physicochemical properties, including their common storage compartment in the neutrophil azurophilic granules, similar protein mass (27–29 kDa), and high isoelectric points (pI 10–12) [9,50,51]. The presence of M2F glycoforms on azurocidin and NE has been reported previously [12,24].

### 2.4. ZIC-HILIC SPE Enrichment of nCG N-Glycopeptides Favors Complex Glycoforms

**Figure 4:**
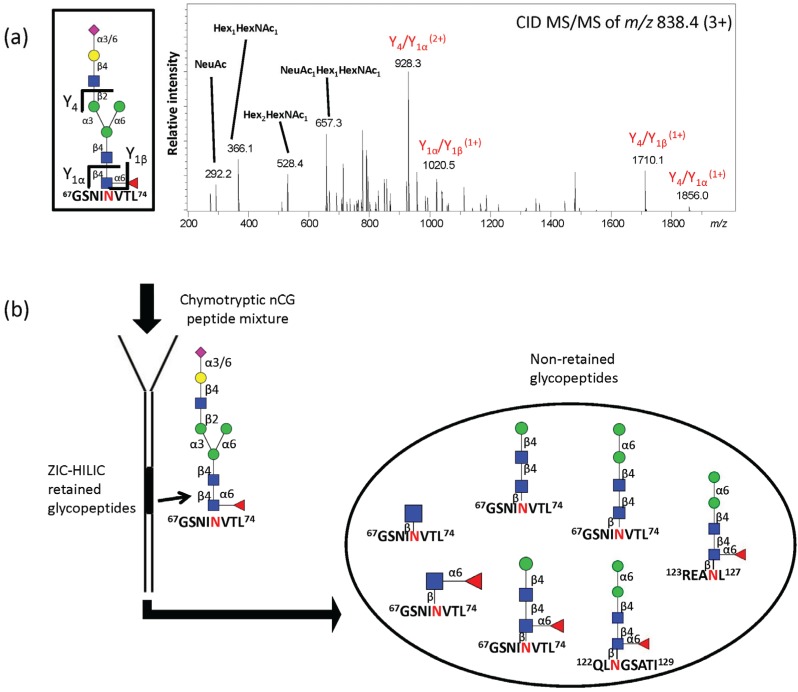
(**a**) RP-LC-CID-MS/MS confirming the structure of the single Asn71-glycopeptide of nCG retained on ZIC-HILIC SPE; (**b**) Schematic illustration showing the population of non-retained chymotryptic *N*-glycopeptides, *i.e.*, Asn71, Asn126, and Asn124 containing glycopeptides of nCG, azurocidin, and NE, respectively, using ZIC-HILIC SPE for glycopeptide enrichment. All glycopeptides were identified by CID- and ETD-MS/MS.

Chymotryptic glycopeptides were enriched using ZIC-HILIC SPE to target the nCG Asn71-glycopeptides carrying the missing glycan structures that were observed in the *N*-glycome, *i.e.*, M0F and complex *N*-glycans. This approach identified the Asn71-glycopeptide (GSNI**N**VTL) carrying the core fucosylated monosialylated complex *N*-glycan with an intact trimannosyl-chitobiose core in the retained ZIC-HILIC fraction (Figure 4a). Interestingly, however, the paucimannosidic and truncated chitobiose core structures carried by the nCG, azurocidin, and NE glycopeptides were, in contrast, identified in the non-retained fraction (Figure 4b). This indicates a requirement of a minimum degree of local hydrophilicity to enable ZIC-HILIC retention, which did not appear to be provided by the paucimannosidic *N*-glycans on these peptides. We have previously reported that M2 and M2F peptides are, at least in part, retained on ZIC-HILIC SPE when using identical stationary and mobile phase conditions *i.e.*, 1% (v/v) trifluoroacetic acid (TFA) as an ion pairing agent [15]. This indicates that the nature of the individual peptide carriers (as well as the mobile and stationary phases [52,53]) significantly influences the ZIC-HILIC retention behavior of these lowly hydrophilic truncated *N*-glycans attached to peptides.

### 2.5. Intact nCG Profiling Maps the Asn71-Glycosylation and Other PTMs

**Figure 5:**
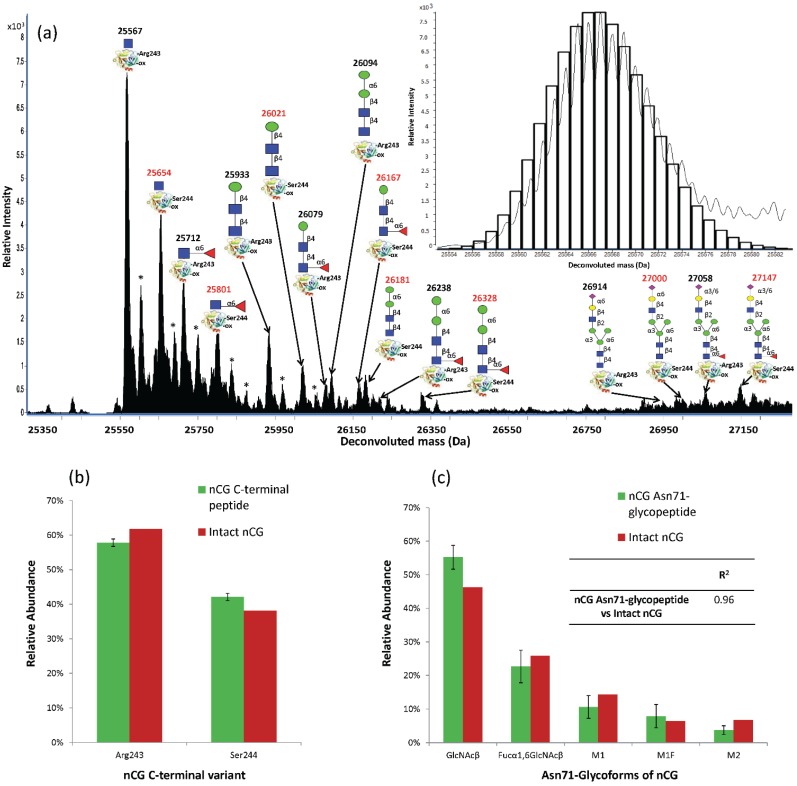
(**a**) QTOF LC-ESI-MS glycoprofiling of intact nCG at relative low fragmentor potential (200 V). The deconvoluted (average, apex) masses and structures of the individual nCG glycoforms are illustrated on the two C-terminal truncation variants of the protein (Arg243- and Ser244-C-terminal nCG variants in black and red, respectively). “-ox” denotes Met152 oxidation. * denotes adduct formation. Insert: Example of the accurate match of the theoretical (black bars) and the observed isotopic distribution of the Arg243-terminating and Asn71-GlcNAcβ and Met152-oxidation containing nCG; (**b**) The ratio of nCG Arg243- and Ser244-truncation variants was confirmed at the peptide level; (**c**) The similar nCG Asn71-glycopeptide and intact nCG glycoform profile, as evaluated by the high correlation coefficient (*R*^2^), confirmed the relative distribution of the Asn71-glycans. The trace Asn71-glycoforms were not included in this quantitative comparison. Data points are plotted as mean ± SD, *n* = 3.

The discrepancy between the *N*-glycome and Asn71-glycopeptide analysis was further investigated by profiling intact nCG using high-resolution/mass accuracy QTOF LC-ESI-MS. Several nCG glycoforms were observed including the truncated chitobiose cores (GlcNAcβ and Fucα1,6GlcNAcβ), paucimannosidic *N*-glycans (M1, M1F, M2, and M2F) and complex structures corresponding to the monoantennary sialylated *N*-glycans (Figure 5a). Interestingly, two C-terminal truncation variants of nCG displaying similar *N*-glycosylation were observed at different relative abundances *i.e.*, the Arg243-(61.9%) and the Ser244-(38.1%) terminating nCG. This was supported by the constant mass deviation of the multiple MS signal pairs (Δm = +87 Da) corresponding to the additional serine residue of the Ser244-variant and the accurate match of the theoretical and experimentally observed isotopic distribution of nCG. The two nCG truncated isoforms were validated at the peptide level by the identification and relative quantitation of the C-terminal ^238^IRTTMR^243^ (57.8% ± 1.1%) and ^238^IRTTMRS^244^ (42.2% ± 1.1%) peptides from the chymotryptic digest (Figure 5b). The Ser244 C-terminal of nCG has been documented before [7,54], but this is, to the best of our knowledge, the first time the additional Arg243 C-terminal on nCG has been defined and quantified.

The intact protein measurements also confirmed that nCG displayed no other modifications except for a single complete oxidation (Δm = +16 Da). The oxidation was localized to Met152 as confirmed by chymotryptic peptide analysis (see Figure S5). The high degree of oxidation of Met152 was supported by its high solvent accessibility (NACCESS score: 123.5) relative to the other more inaccessible methionine residues on the maturely folded nCG (*i.e.*, Met35 NACCESS score: 5.1, Met110 NACCESS score: 0, Met242 NACCESS score: 27.1). Oxidation of methionine residues of nCG has been reported to be modulated by myeloperoxidase as a mechanism to regulate the proteolytic activity of nCG [55]. The peptide analysis also confirmed that specific glutamine (Gln163) and asparagine (Asn208) residues were found to be deamidated. The deamidation may have been introduced naturally within the cell upon biosynthesis or artificially due to sample handling and/or during the MS/MS analysis [56]. The intact nCG glycoprofile accurately reflected the analysis of the Asn71-glycopeptide profile (*R*^2^ = 0.96) rather than the *N*-glycan profile that was influenced by the presence of the other interfering neutrophil glycoproteins (*R*^2^ = 0.38), thereby validating the correct Asn71-glycoprofile of nCG (Figure 5c). In a previous study by Watorek and coworkers, M2F and biantennary disialylated complex *N*-glycans were the only structures documented on nCG [12]. However, we did not observe biantennary disialylated complex *N*-glycans in our analysis, possibly due to differences in purification and the applied analytical methods. Table 1 provides a semi-quantitative overview of the site-specific *N*-glycosylation of nCG, azurocidin, and NE observed in this study.

### 2.6. Establishing Asn71 Occupancy Level and N-Glycosidase F-Resistant Glycoforms of nCG

Initially, intact nCG was profiled under high fragmentor potential (>300 V), which suggested that a significant proportion of the nCG molecules appeared without a conjugated Asn71-glycan (Figure S6). Various fragmentor voltages were applied (150–400 V) to determine if the non-glycosylated nCG variants were indeed MS artefacts caused by in-source/post-source fragmentation due to the relative high fragmentor potential, rather than being naturally occurring proteoforms. It was observed that under milder MS conditions (200 V), the non-glycosylated nCG variants were absent. The complete Asn71-glycosylation site occupancy and the glycoform distribution were validated by excellent agreement of the intact glycoprotein and glycopeptide profiles (described above). We have previously validated that *N*-glycopeptides undergo no detectable fragmentation under the regular LC-ESI-MS/MS acquisition conditions and no non-glycosylated chymotryptic Asn71-peptides were observed in the chymotryptic peptide mixture, suggesting full occupancy of Asn71. Non-glycosylated peptides have been previously documented to ionize preferentially over the larger *N*-glycosylated peptides [30], thereby excluding a potential bias towards the glycosylated nCG in this evaluation. Site occupancy can also typically be evaluated using the non-glycosylated to de-*N*-glycosylated peptide ratio after *N*-glycosidase F treatment, due to the more similar ionization properties of these two species [30]. However, it was observed that the GlcNAcβ and Fucα1,6GlcNAcβ carbohydrate moieties were not removed from the peptide carriers by *N*-glycosidase F even under favorable enzyme concentrations, while, as expected, the paucimannosidic and complex *N*-glycans were completely removed from the same peptide during this treatment (see Figure S7). The *N*-glycosidase F-resistance of truncated chitobiose cores, which has been reported previously [44], not only excludes the possibility of performing the site occupancy based on the non- to de-*N*-glycosylated peptide ratio, but also masks these truncated structures in the regular *N*-glycosidase F-based *N*-glycome profiles.

### 2.7. The Proximal, but Short, Asn71-Glycans are not Obstructing the Active Site of nCG

**Figure 6:**
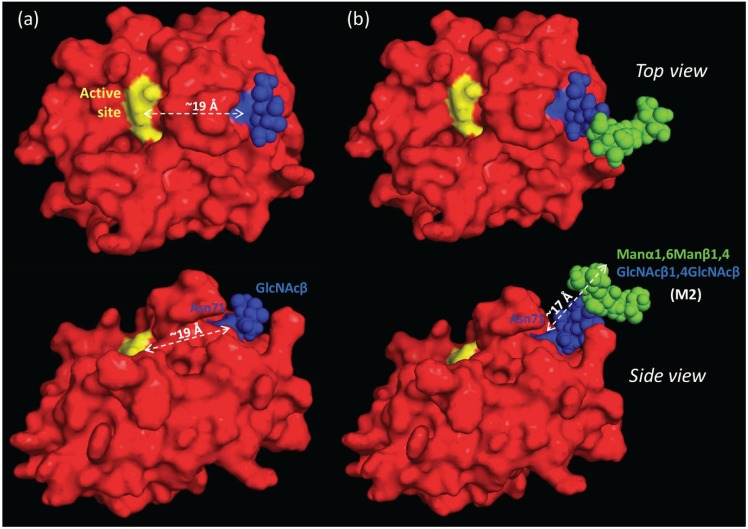
Top and side view of nCG illustrating the proximity (~19 Å) of the active site to the Asn71-glycosylation site conjugated with (**a**) GlcNAcβ and (**b**) M2 (Manα1,6Manβ1,4GlcNAcβ1,4GlcNAcβ). Color scheme: yellow denotes His64, Asp108, and Ser201 forming the active site of nCG; blue denotes the Asn71-glycosylated residue and the conjugated GlcNAc residues; and green denotes mannose residues. The approximate height of the M2 *N*-glycan (~17 Å) is illustrated.

Consulting a high-resolution three-dimensional (3D) structure of nCG [8] revealed that although Asn71 is proximal to the active site of nCG (~19 Å), the conjugated *N*-glycans are unlikely to interfere directly with the catalytic activity of the protein due to their truncated nature (e.g., the height of GlcNAcβ is ~5 Å and M2 is ~17 Å) (see Figure 6). Only the low abundant elongated monoantennary complex sialo-*N*-glycans (height: ~27 Å) would theoretically be able to sterically interfere with the accessibility to the active site (see Figure S8). However, the presence of a bulky protein surface domain separating the Asn71-glycosylation site and the active site, and the fact that Asn71-conjugated *N*-glycans appear to be pointing away from the active site when using the default torsion angles to assess its presentation on the protein surface, makes any such interference unlikely. It was previously concluded that Asn71-glycosylation is not essential for the enzymatic activation and granule sorting of nCG [13]. Thus, the potential modulatory roles of Asn71-glycosylation on the nCG activity, its structural conformation, or any protein-independent functions of the carbohydrate moiety remain to be determined.

### 2.8. Spatial Considerations of nCG Structure Advance our Understanding of the Unconventional Asn71-Glycosylation

The Asn71-glycosylation site displayed medium-low solvent accessibility (NACCESS score: 44.1) upon assessment of the 3D structure of maturely folded nCG. We have previously demonstrated that the solvent accessibility of *N*-glycosylation sites of maturely folded glycoproteins correlates with the degree of *N*-glycan processing at that site and, thus, determines multiple glycan features including the glycan type, core fucosylation, and branching [57]. We have also demonstrated that subcellular-specific *N*-glycosylation appears to be related to differential solvent accessibilities of the *N*-glycosylation sites of proteins residing in different subcellular compartments [58]. Asn sites displaying paucimannosylation were shown to have higher solvent accessibility in comparison to the relatively inaccessible high mannose containing sites (NACCESS score typically 0–40), indicating that the truncated paucimannosidic proteins are generated by exposure to glycan processing by hydrolases (*i.e.*, hexosaminidases and mannosidases) [15]. No protein features other than this differential accessibility and the subcellular localization have so far been correlated with the expression of these unusual paucimannosidic structures that are abundant in the azurophilic granules of neutrophils.

The medium-low solvent accessibility of Asn71 on nCG implies that the conjugated *N*-glycan intermediates were only partially available for processing during biosynthesis to the highly truncated GlcNAcβ and Fucα1,6GlcNAcβ glycoforms. In comparison, the Asn-sites of the M2F-carrying azurocidin and NE were found to be highly accessible (NACCESS score: 80.8–103.1). Hence, solvent accessibility alone does not explain the extreme Asn71-glycan truncation on nCG, but is congruent with the relatively low degree of core fucosylation (~30%) of nCG *N*-glycans. We are currently investigating the presence of other protein features required for the generation of paucimannose, chitobiose core, and GlcNAcβ-/Fucα1,6GlcNAcβ-containing glycoproteins.

### 2.9. Subcellular-Specific N-Glycosylation of nCG in Human Neutrophils

Using site-specific profiling of enriched tryptic glycopeptides [15] and subcellular-specific libraries of neutrophil glycoproteins [16], we previously suggested that paucimannosidic and monoantennary complex (NeuAc_1_Gal_1_Man_3_GlcNAc_3_Fuc_0-1_) *N*-glycans are carried by glycoproteins localizing to different subcellular compartments of the human neutrophil *i.e.*, azurophilic and the specific granules, respectively [15]. This was supported by the pronounced spatio-temporally regulated biosynthesis of glycoproteins in neutrophils generating compartment-specific *N*-glycosylation during the maturation of these immune cells in the bone marrow [15]. This process is referred to as “targeted-by-timing” and thought to be responsible for the compartment-specific *N*-glycosylation of neutrophils [15,59,60]. Thus, the observation of both paucimannosidic and monoantennary complex Asn71-glycans (as well as the further truncated chitobiose core structures) on nCG was, at first, rather surprising. However, nCG has in fact been found to reside in the azurophilic (~75%), in the specific granules (~15%), and in other compartments [16], thereby supporting the feature of subcellular-specific *N*-glycosylation on the same site of the same glycoprotein in different compartments within the human neutrophil. Isolation and structural analysis of nCG from the individual subcellular compartments of the human neutrophil are needed to confirm this proposed relationship. Although few other studies have reported the presence of truncated chitobiose glycoproteins from multiple cellular origins in mammals and invertebrates [45,46,47,48,49], indicating that the human neutrophil may be just one amongst many biological systems displaying these structures, their exact subcellular localization and potential functions remain to be investigated.

## 3. Experimental Section

### 3.1. Origin and Initial Handling of nCG

Purified human nCG (UniProt accession number: P08311) originating from resting neutrophils isolated from whole blood of a pool of healthy individuals was purchased from Lee BioSolutions, product number: 186-10 (St Louis, MO, USA). The purity was assessed by sodium dodecyl sulfate polyacrylamide gel electrophoresis (SDS-PAGE) (Bio-Rad, Sydney, Australia) upon arrival (see Figure S1), and the protein was aliquoted (20 µg) and stored at −20 °C until use.

### 3.2. N-Glycan Release and Handling

Proteolytical inactivation of nCG was achieved using 1.5 mM PMSF for 90 min at 22 °C (see Figure S1). Subsequently, nCG was reduced using 10 mM dithiothreitol (final concentration) for 45 min at 56 °C and alkylated using 25 mM iodoacetic acid (final concentration) for 30 min in the dark at 22 °C. nCG was then analyzed by SDS-PAGE under reducing and denaturing conditions, using a 4–15% gradient gel at 120 V for 1 h at 22 °C. Subsequently, the protein was transferred to a primed 0.45-μm PVDF membrane (Millipore, Bayswater, Australia) using a trans-blot turbo transfer system (BioRad) and stained with Direct Blue (Sigma-Aldrich, Castle Hill, Australia). The resulting 24–26 kDa gel band was excised and washed in separate wells in a flat bottom polypropylene 96-well plate (Corning Life Sciences, Corning, NY, USA). *N*-glycans were released and prepared from the membrane as previously described [20]. In brief, 3.5 U *N*-glycosidase F (*Flavobacterium meningosepticum*, Roche, Castle Hill, Australia) was used per 10 μg protein in a 10 μL water/well for 16 h at 37 °C. Released *N*-glycans were incubated with 100 mM ammonium acetate (pH 5) for 1 h at 22 °C. Glycan reduction was performed with 1 M sodium borohydride in 50 mM potassium hydroxide for 3 h at 50 °C, followed by glacial acetic acid quenching of the reaction. Dual desalting steps were performed in micro-SPE formats with strong cation exchange/C18 (where *N*-glycans are not retained) and PGC (where *N*-glycans are retained) stationary phases, respectively. Elution from the PGC-SPE columns was performed with 40% (v/v) acetonitrile (ACN) containing 0.1% (v/v) TFA and dried by vacuum centrifugation. Fractions were taken up in 10 μL of water and analyzed using PGC-LC-MS/MS.

### 3.3. Exoglycosidase Treatment of Released N-Glycans

Aliquots of released and reduced *N*-glycans were digested for 16 h at 37 °C with multiple exoglycosidases in a final reaction volume of 10 μL. Specifically, digestions were performed with α2,3/6/8-unspecific sialidase (2 U) from *Arthrobacter ureafaciens* and the α2,3-specific sialidase (2 U) from *Streptococcus pneumonia* in 50 mM sodium phosphate, pH 6 buffer. The α1,2/3- > α1,6-linkage-preferring Jack bean meal α-mannosidase (2 U) was performed in 20 mM sodium acetate, 2 mM zinc chloride, pH 5 buffer. All enzymes were purchased from Prozyme (Hayward, CA, USA). The exoglycosidases were removed by retention on the strong cation exchange/C18 and thus separated from the glycans in the sample preparation prior to PGC-LC-MS/MS.

### 3.4. PGC-LC-ESI-MS/MS-Based N-Glycome Profiling

*N*-glycans were analyzed by capillary LC-MS/MS (Agilent 1260 Infinity) using an ESI ion trap mass spectrometer (LC/MSD Trap XCT Plus Series 1100, Agilent Technologies, Melbourne, Australia). Samples were injected onto a PGC-LC capillary column (Hypercarb KAPPA, 5 μm particle size, 200 Å pore size, 180 μm inner diameter x 100 mm length, Thermo Scientific, Scoresby, Australia) and separation of *N*-glycans was carried out over a linear gradient of 0–45% (v/v) ACN/10 mM ammonium bicarbonate for 85 min at a constant flow rate of 2 μL/min. The sample injection volume was 3 μL. The acquisition range was *m/z* 200–2200. The acquisition was performed in negative ionization polarity in a data-dependent acquisition manner where the top two most abundant precursors in each full scan spectrum were selected for MS/MS using CID. The mass spectrometer was calibrated using a tune mix (Agilent Technologies). Mass spectra were viewed and analyzed using DataAnalysis v4.0 (Bruker Daltonics, Melbourne, Australia). Glycoworkbench v1.2.4 assisted in the annotation and visualization of the *N*-glycan structures [61].

### 3.5. In-Solution Glycopeptide Generation, Enrichment, and Deglycosylation

nCG was proteolytically inactivated, reduced, and alkylated as described above. In-solution proteolytic digestion was carried out using bovine pancreas chymotrypsin (sequence grade, Roche) at 1:20 enzyme/substrate ratio (w/w) for 18 h at 25 °C in aqueous 50 mM ammonium bicarbonate, pH 8.4. The resulting chymotryptic peptide mixture was aliquoted, dried, and stored at −20 °C until use. Glycopeptide enrichment from the chymotryptic peptide mixture was performed by redissolving nCG in 10 μL of 80% (v/v) ACN in aqueous 1% (v/v) TFA using a custom-made ZIC-HILIC SPE equipped with a C18 disc (Millipore) to allow packing of the ZIC-HILIC material in the micro-column. The stationary phase consisted of ZIC-HILIC resin (10 μm particle size, 200 Å pore size, Sequant/Merck, Solna, Sweden). The columns were prepared as previously described [53]. In brief, the ZIC-HILIC resin was packed in the column (height: 5–10 mm, column volume: ~0.5–1 μL) and equilibrated in 50 μL mobile phase consisting of 80% (v/v) ACN/1% (v/v) TFA. Samples were loaded repeatedly onto the column in successive rounds before washing the column twice with 50 μL of the mobile phase. The enriched glycopeptides were then eluted using 2 × 50 μL 1% (v/v) TFA. An additional elution step with 50 μL of 80% (v/v) ACN/1% (v/v) TFA was carried out to ensure all glycopeptides were eluted from the C18 base of the column. Fractions were dried and taken up in 10 μL of 0.1% (v/v) formic acid (FA) for multiple LC-MS/MS injections. De-*N*-glycosylation was performed by redissolving aliquots of the chymotryptic peptide mixtures in 50 μL water and incubation with 10 U *N*-glycosidase F (Roche) for 16 h at 37 °C.

### 3.6. LC-MS/MS-Based N-Glycopeptide Analysis

nCG peptide mixtures were analyzed by ESI-MS/MS in positive polarity using an HCT 3D ion trap (Bruker Daltonics) coupled to an Ultimate 3000 LC (Dionex, Australia). The samples were loaded directly onto a C18 column (Proteocol HQ303, 300 μm inner diameter x 10 cm length, 3 μm particle size, 300 Å pore size, SGE, Australia). The column was equilibrated in 100% solvent A consisting of aqueous 0.1% (v/v) FA and a gradient up to 30% (0.5%/min slope) and a second gradient up to 60% (4.2%/min slope) of solvent B consisting of 0.1% (v/v) FA in ACN for 60 min and 7 min, respectively, before washing the column in 80% solvent B for 10 min and re-equilibration in the starting condition. A constant flow rate of 5 μL/min was used. The chymotryptic peptide mixture was analyzed with and without ZIC-HILIC-SPE-based glycopeptide enrichment in technical triplicates. Two injections (5 μL/injection) was performed in separate runs using the following setups: (1) LC-MS/MS analysis, where an MS full scan (*m*/*z* 300–2200; scan speed: 8100 *m*/*z*/s) was followed by a data-dependent fragmentation of the three most abundant signals using CID; and (2) LC-MS/MS analysis, where a MS full scan (*m*/*z* 400–1800) was followed by an ETD event of the two most abundant signals in the full scan. The ETD settings were as follows: ion count control reactant target ETD: 600,000, reactant accumulation time: 4–20 ms (≤ 200 ms), reaction time: 150 ms. Both CID- and ETD-LC-MS/MS were used for site-specific characterization of the nCG glycoforms. The mass accuracy of the mass spectrometer was calibrated using a tune mix (Agilent Technologies) prior to acquisition. Mass spectra were viewed and analyzed using DataAnalysis v4.0 (Bruker Daltonics) and analysis was performed using GPMAW v10.0 (Lighthouse, Odense, Denmark) [62] using the protein sequence of nCG (UniProt accession number: P08311), azurocidin (UniProt accession number: P20160) and NE (UniProt accession number: P08246).

### 3.7. Intact nCG Profiling

Intact nCG glycoprotein (1 μg) was analyzed by ESI-MS in positive ion polarity mode using a high-resolution/high mass accuracy QTOF 6538 mass spectrometer (Agilent Technologies) coupled to a capillary LC (Agilent 1260 Infinity). nCG was loaded directly onto a C4 column (Proteocol C4Q, 3 μm particle size, 300 Å pore size, 300 μm inner diameter x 10 cm length, SGE, Australia). The column was equilibrated in identical mobile phases as for the C18 column (described above) with a gradient up to 60% (v/v) (2%/min slope) of solvent B before washing the column in 99% (v/v) solvent B for 10 min and re-equilibration in the starting condition. A constant flow rate of 5 μL/min was used. One-microliter injections were used. Various fragmentor potentials (150–400 V) were tested in separate runs using the following MS settings in high-resolution (4 GHz) mode: MS full scan (*m*/*z* 400–2500), drying gas temperature 300 °C, drying gas flow rate 8 L/min, nebulizer pressure 10 psig, capillary potential 4300 V, skimmer potential 65 V. The mass accuracy of the mass spectrometer was calibrated using a tune mix (Agilent Technologies) prior to acquisition. An internal mass calibration sample was infused continuously during the LC-MS run to allow accurate and automated in-spectrum mass calibration. Generally, mass accuracies better than 2 ppm were achieved. Mass spectra were viewed and analyzed with MassHunter workstation vB.06 (Agilent Technologies).

### 3.8. Profiling nCG N-Glycans, N-Glycopeptides, and Intact Glycoprotein

The detailed nCG *N*-glycans structures were manually determined by their monoisotopic masses, CID-MS/MS fragmentation patterns, and their relative and absolute retention times based on the PGC-LC-MS/MS *N*-glycome data (see Figure S3 and Table S1 for data supporting the *N*-glycan characterization). The relative distribution of the individual *N*-glycans was estimated based on the relative peak area of the extracted ion chromatograms (EICs) of all observed charge states of each *N*-glycan against the total EIC peak area of all observed *N*-glycans.

The structure of the observed *N*-glycopeptides were manually determined by their monoisotopic masses, CID- and ETD-MS/MS fragmentation patterns and retention times based on the RP-LC-MS/MS analysis of chymotryptic *N*-glycopeptides (see Figure S4 and Table S2 for data supporting the *N*-glycopeptide characterization).

Chymotryptic Asn71-glycopeptides of nCG eluted around 39 ± 1 min (Figure S7). LC-MS/MS spectral data from both the non-enriched and ZIC-HILIC-SPE-enriched glycopeptide fractions were used for site-specific characterization of Asn71-glycosylation. Only the non-enriched peptide mixtures were used for quantitative glycoprofiling. Briefly, the distribution of the glycoforms and the site occupancy level were estimated from the relative EICs area of all observed charge states of Asn71-glycopeptides by assuming equal ionization efficiencies of these related molecular species [30,63].

Intact nCG was profiled by deconvoluting the obtained spectra using the default settings in the maximum entropy algorithm. The relative abundance of the observed glycoforms was determined from their relative EICs area as calculated using BioConfirm in MassHunter workstation vB.06 (Agilent Technologies).

### 3.9. Glycoprotein Modeling

The 3D protein structure of nCG obtained by X-ray crystallography (PDB accession number: 1CGH) was used for modeling and solvent accessibility determination [8]. This particular 3D structure was chosen over other available structures due to its high sequence coverage of nCG, spatial resolution and the natural source of the protein, which was purified directly from human neutrophils. Visualization and distance measurements were performed using PyMOL Molecular Graphic System, v1.3 (Schrödinger, LLC) and RasMol v2.7.5, respectively. *N*-glycans were modeled on Asn71 of nCG *in silico* using the default torsion angles provided by Glyprot [64]. The solvent accessibilities of Asn71 and the individual methionine residues of nCG were determined using NACCESS, a solvent accessibility determination program [65]. The atomic accessible areas (van der Waal’s interactions) were measured in absolute arbitrary units by rolling a 5 Å probe on the protein surface of nCG [66].

### 3.10. Statistics

Data points collected as technical triplicates were presented as mean ± standard deviation (SD). Statistical regression analyses were carried out using Microsoft Excel.

## 4. Conclusions

Developments in LC-MS/MS-based glycoproteomics [67,68] and “top down” glycoprofiling of intact glycoproteins [33,69] are providing us with powerful tools that undoubtedly will advance our understanding of many structural aspects of glycobiology. However, it remains important to stress that these analytical tools are still relatively immature and, at present, incapable by themselves of providing information on all the levels of structural heterogeneity displayed by glycoproteins, even those with a single site of glycosylation, as clearly illustrated in this study. Therefore, it is still necessary to apply an array of technologies, in particular when mapping proteins displaying the described truncated *N*-glycosylation.

Herein, detailed site-specific Asn71-glycoprofiling using glycomics, glycopeptide, and intact glycoprotein MS analysis has documented that nCG carries unconventional *N*-glycosylation including a truncated chitobiose core (GlcNAcβ and Fucα1,6GlcNAcβ), as well as paucimannosidic (M1, M1F, M2, and M2F) and monoantennary sialo *N*-glycans. This structural library of the nCG molecular heterogeneity further confirms the unconventional and subcellular-specific *N*-glycosylation in the human neutrophil. The functional consequences of the unique glycosylation utilized by human neutrophils remain to be elucidated and we are currently investigating the functional role of the unconventional Asn71-glycosylation of nCG by building on the observations provided here.

## Supplementary Files

Supplementary File 1
