# Supplementary material for: Complementary LC-MS/MS-Based N-Glycan, N-Glycopeptide, and Intact N-Glycoprotein Profiling Reveals Unconventional Asn71-Glycosylation of Human Neutrophil Cathepsin G
Source: Biomolecules. 2015 Aug 12;5(3):1832–54. doi: 10.3390/biom5031832 (PMC4598777; doi:10.3390/biom5031832)

Supplementary file for

Complementary LC-MS/MS based *N*-glycan, *N*-glycopeptide and intact *N*-glycoprotein profiling reveals unconventional Asn71-glycosylation of human neutrophil cathepsin G

Ian Loke, Nicolle H. Packer, Morten Thaysen-Andersen *

Department of Chemistry and Biomolecular Sciences, Macquarie University, North Ryde, NSW 2109, Australia. *Author to whom correspondence should be addressed; E-Mail: morten.andersen@mq.edu.au (M.T.-A.); Tel.: +61-2-9850-7487; Fax: +61-2-9850-6192.

Table S1: Overview of the glycan numbers, masses and monosaccharide compositions of the *N*-glycans released from the nCG protein preparation.
Figure S1: Assessment of nCG purity and autoproteolytic activity with and without PMSF treatment using gel electrophoresis under reducing and denaturing conditions.
Figure S2: Separation of sialo-isomers of three types of monoantennary complex *N*-glycans using PGC-LC-CID-MS/MS.
Figure S3: Annotated PGC-LC-ESI-CID-MS/MS spectra of all observed *N*-glycans released from the protein preparation of nCG.
Figure S4: Annotated RP-LC-ESI-CID/ETD-MS/MS spectra of all *N*-glycopeptides identified from the chymotryptic peptide mixture of the nCG protein preparation.
Figure S5: Presence of oxidised and absence of non-oxidised Met152 on a chymotryptic peptide of nCG as evaluated using RP-LC-ESI-ETD-MS/MS.
Figure S6: Comparison of high and low fragmentor potential when profiling intact nCG using high resolution QTOF-ESI-MS.
Figure S7: *N*-glycosidase F-resistance of truncated chitobiose core Asn71-glycopeptides of nCG.
Figure S8: 3D modelling of trimannosyl-chitobiose core monoantennary core fucosylated α2,6-monosialylated *N*-glycan on Asn71 of nCG.

Supplementary Table S1. Overview of the glycan numbers, masses and monosaccharide compositions of the *N*-glycans released from the nCG protein preparation.

Glycan #	RT (min)	*m/z*	Charge (Z)	Obs. Mass (M, Da)	Theo mass (M, Da)	Hex	HexNAc	dHex	NeuAc	Type
1	33.9	571.3	1-	572.3	572.2		2	1		Core fucosylated chitobiose core (M0F)
2	31.3	587.5	1-	588.5	588.2	1	2			Paucimannose (M1)
3	40.9	733.3	1-	734.3	734.3	1	2	1		Core fucosylated paucimannose (M1F)
4	36.1	749.3	1-	750.3	750.3	2	2			Paucimannose (M2)
5	44.7	895.3	1-	896.3	896.3	2	2	1		Core fucosylated paucimannose (M2F)
6	51.0	1057.4	1-	1058.4	1058.4	3	2	1		Core fucosylated paucimannose (M3F)
7a	45.2	775.3	2-	1552.6	1552.5	3	3	1	1	Bimannosyl-chitobiose core monoantennary core fucosylated α2,6-monosialylated
7b	51.5	775.3	2-	1552.6	1552.5	3	3	1	1	Bimannosyl-chitobiose core monoantennary core fucosylated α2,3-monosialylated
8	42.2	783.6	2-	1569.2	1568.5	4	3		1	Trimannosyl-chitobiose core monoantennary α2,6-monosialylated
9a	47.5	856.4	2-	1714.8	1714.6	4	3	1	1	Trimannosyl-chitobiose core monoantennary core fucosylated α2,6-monosialylated
9b	55.1	856.4	2-	1714.8	1714.6	4	3	1	1	Trimannosyl-chitobiose core monoantennary core fucosylated α2,3-monosialylated

Supplementary Figure S1. Assessment of the nCG purity and autoproteolytic activity with and without PMSF treatment using gel electrophoresis under reducing and denaturing conditions. Gel stained with Coomassie Brilliant Blue. *Separate gel.

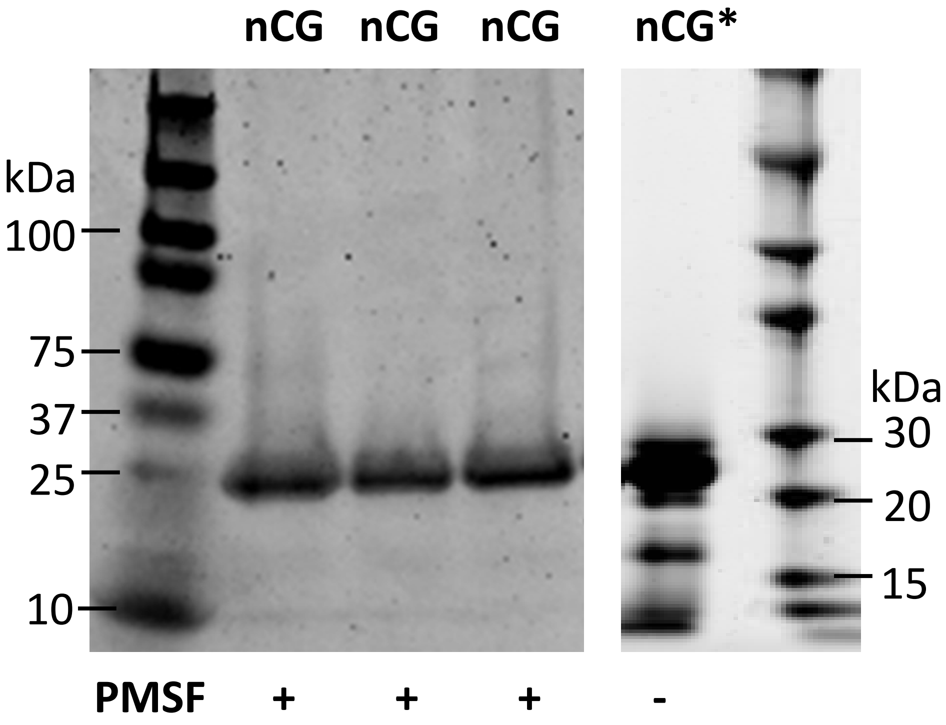

Supplementary Figure S2. Separation of sialo-isomers of three types of monoantennary complex *N*-glycans using PGC-LC-CID-MS/MS [23]. Symbols are used according to the Consortium for Functional Glycomics / Essentials of Glycobiology notation.

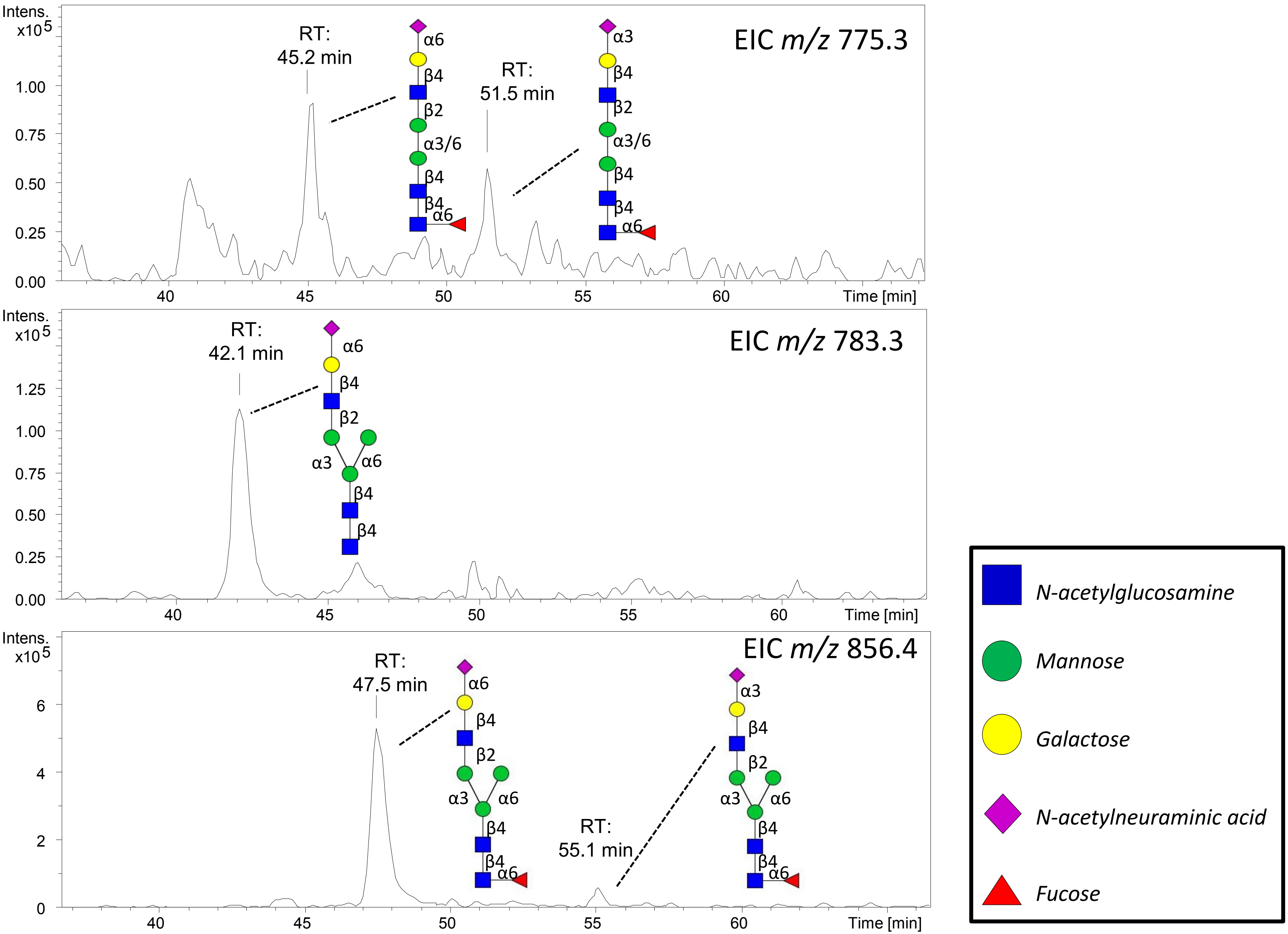

Supplementary Figure S3. Annotated PGC-LC-ESI-CID-MS/MS spectra of all observed *N*-glycans released from the protein preparation of nCG. Fragmentation of the glycans were annotated according to the Domon-Costello nomenclature [34]. All released glycans were in their reduced (alditol) prior to analysis.

Glycan # 1

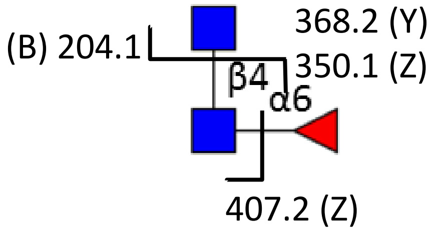

M0F

Observed *m/z* 571.3 (1-), RT: 33.9 min
$[M-H]^-$ 571.3

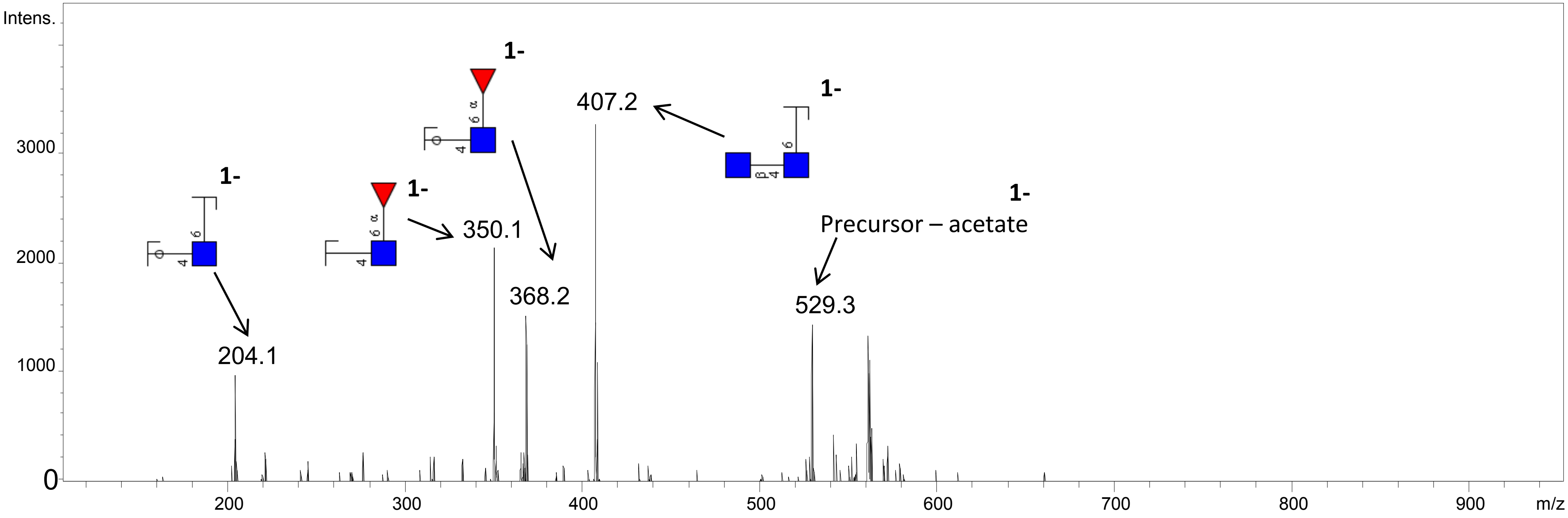

Glycan # 2

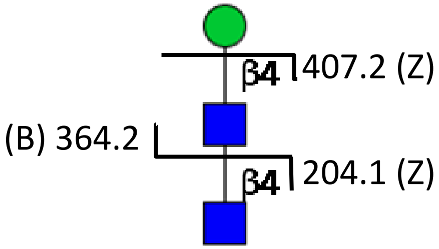

M1

Observed *m/z* 587.3 (1-), RT: 31.3 min
$[M-H]^-$ 587.3

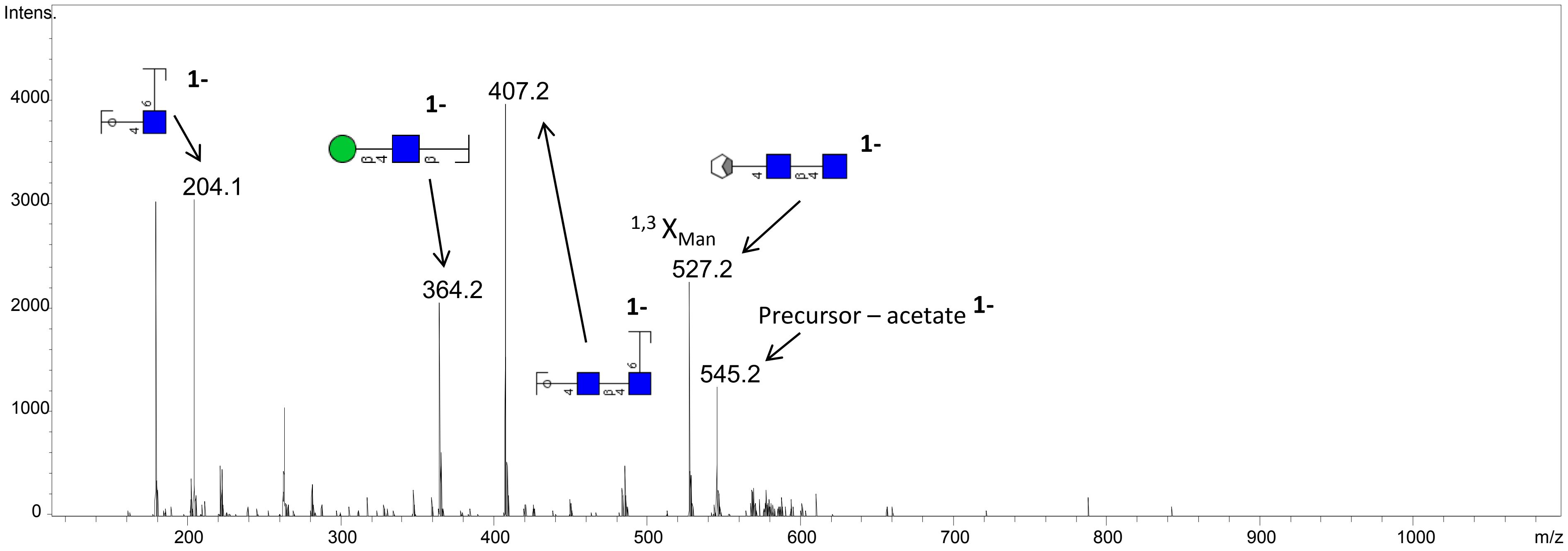

Glycan # 3

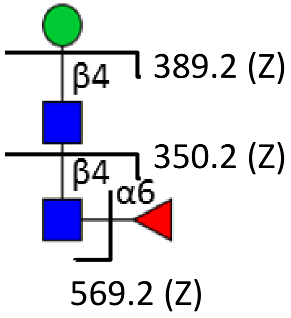

M1F

Observed *m/z* 733.3 (1-), RT: 40.9 min

$[M-H]^-$ 733.3

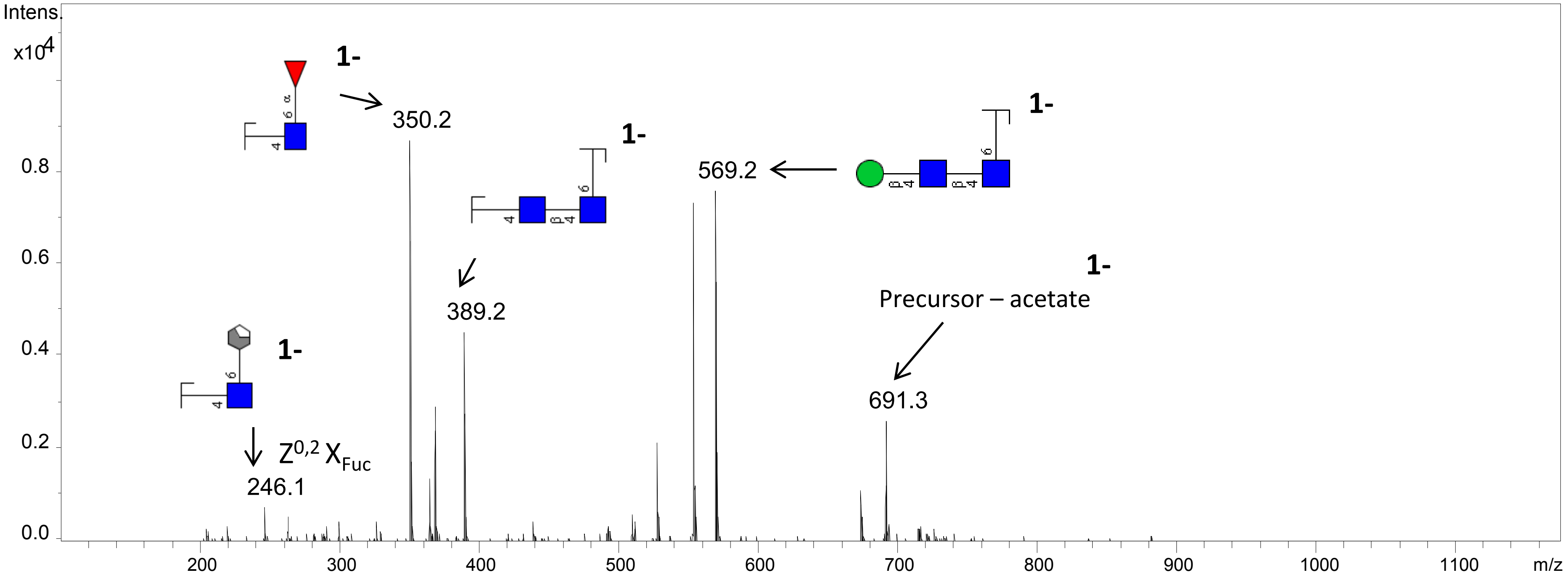

Glycan # 4

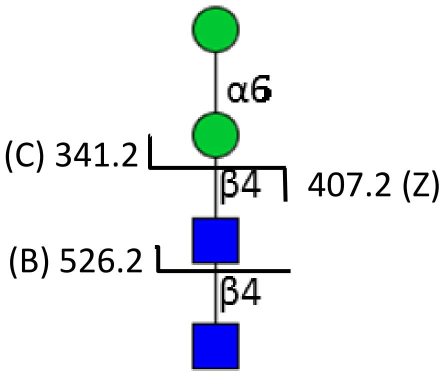

M2

Observed *m/z* 749.3(1-), RT: 36.1 min
$[M-H]^-$ 749.3

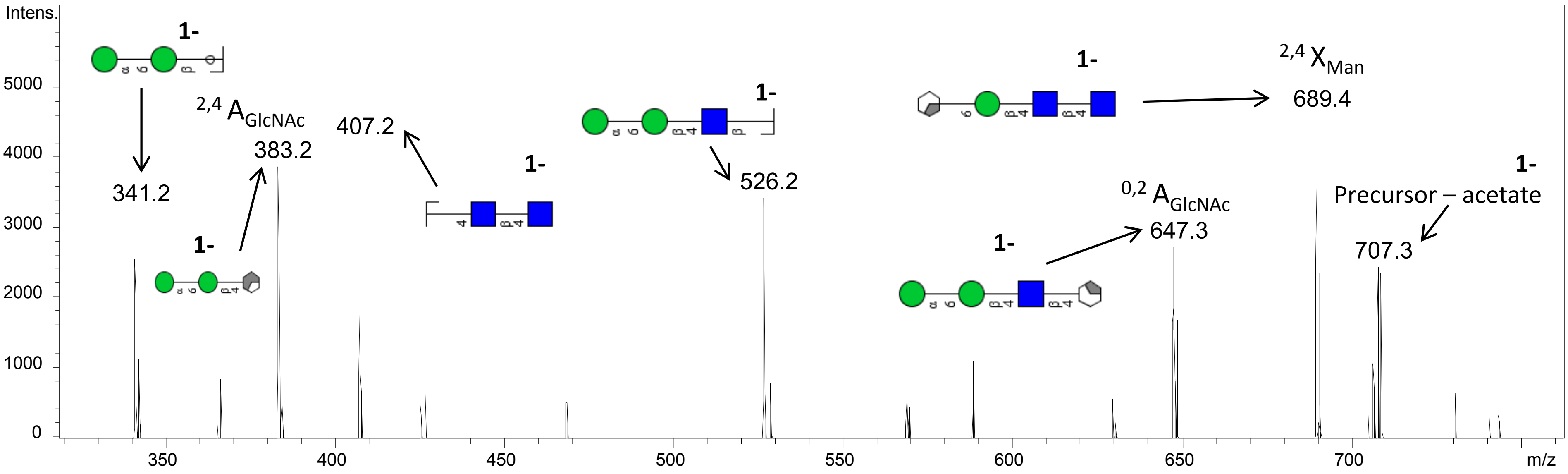

Glycan # 5

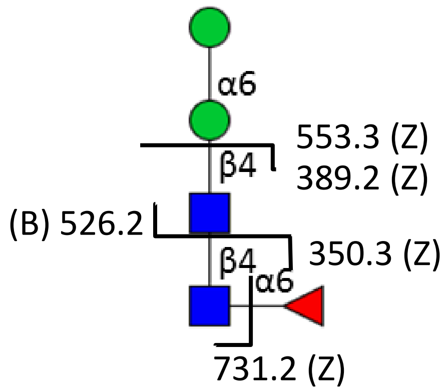

M2F

Observed *m/z* 895.4 (1-), RT: 44.7 min

$[M\text{-}H]^-$ 895.4

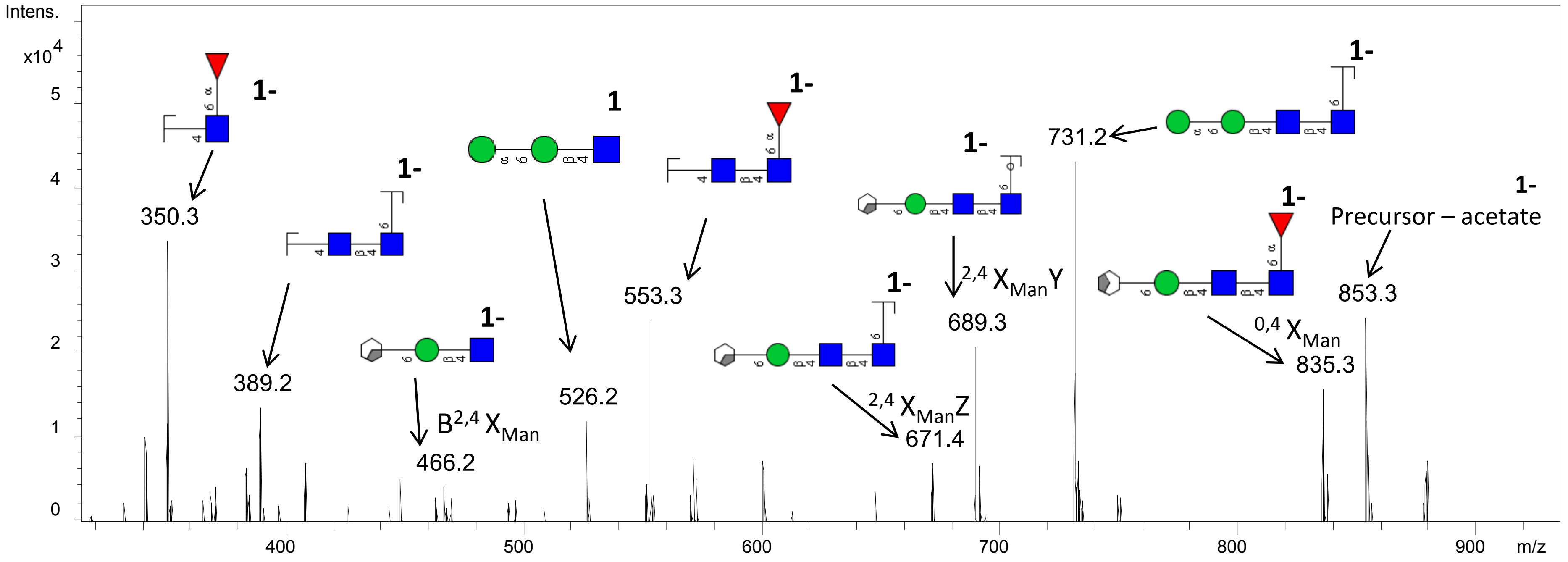

Glycan # 6

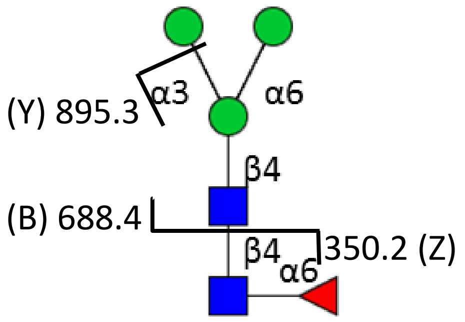

M3F

Observed *m/z* 1057.4 (1-), RT: 51 min

$[M\text{-}H]^-$ 1057.4

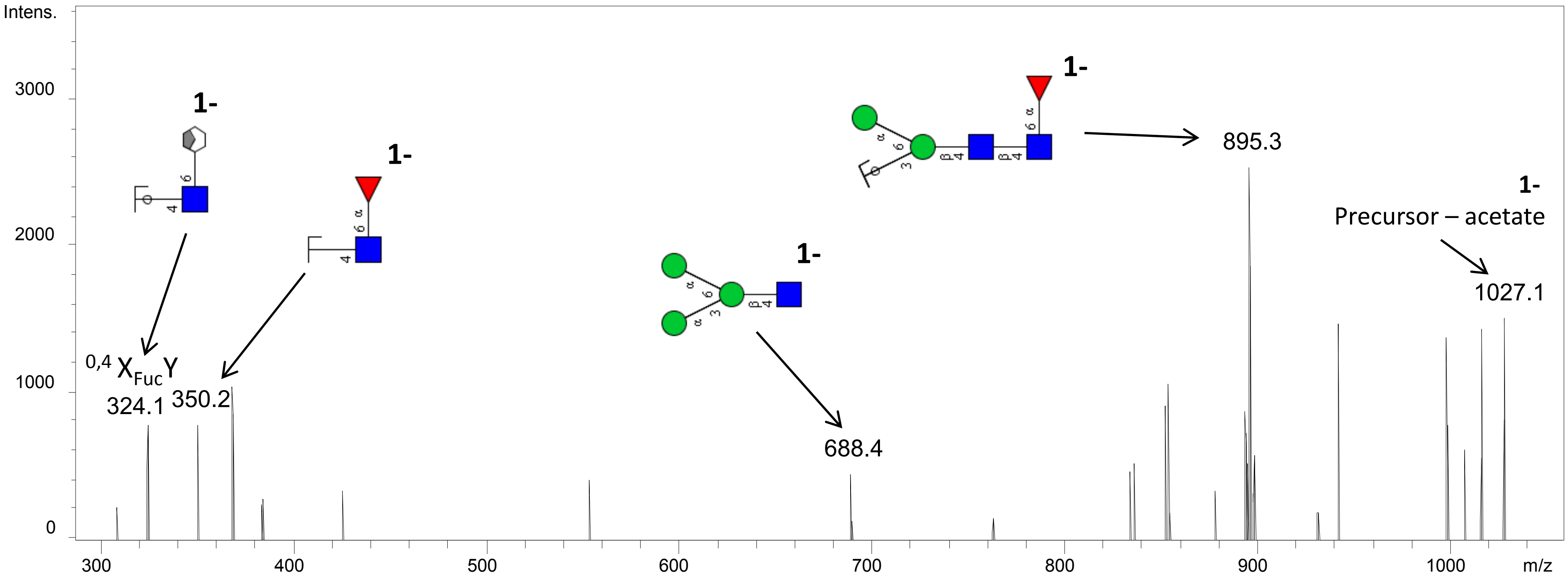

Glycan # 7a

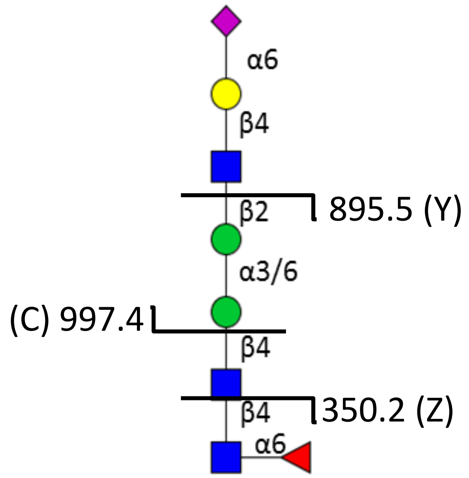

Observed *m/z* 775.3 (2-), RT: 45.2 min

$[M-H]^-$ 1552.6

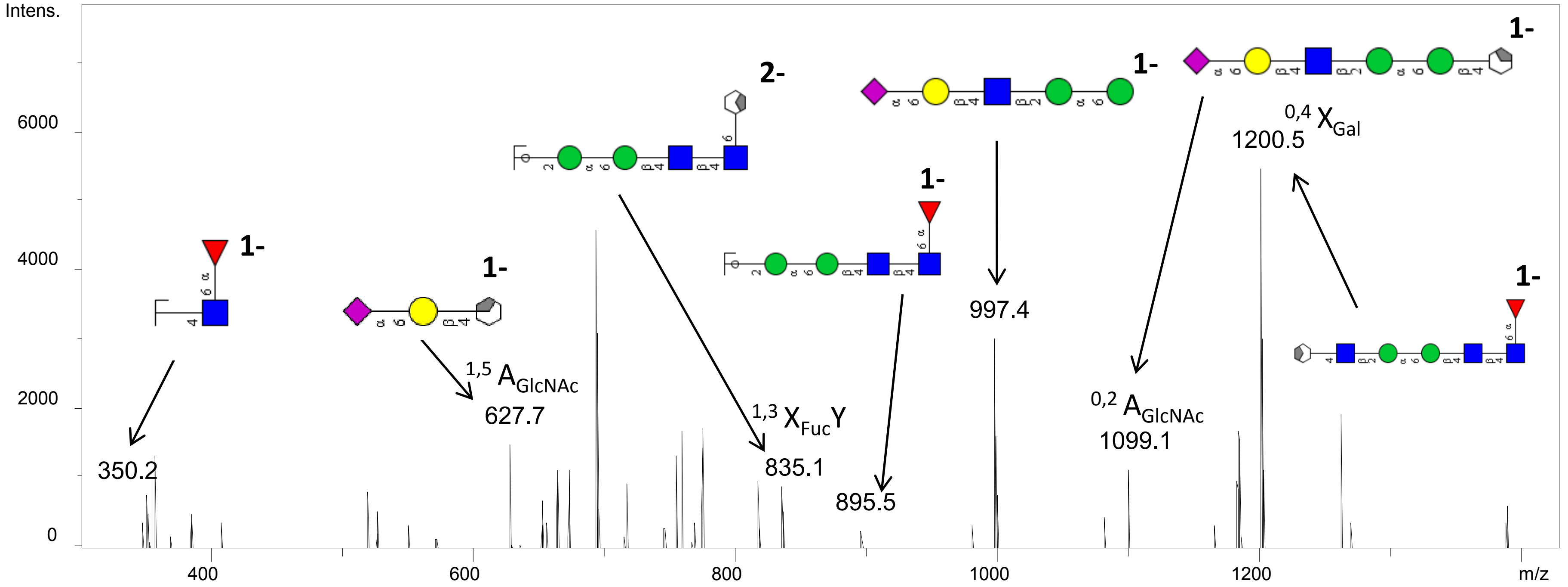

Glycan # 7b

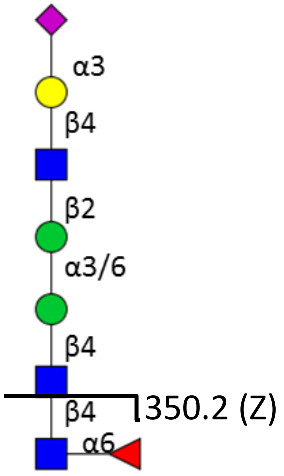

Observed *m/z* 775.3 (2-), RT: 51.5 min

[M-H]$^-$ 1552.6

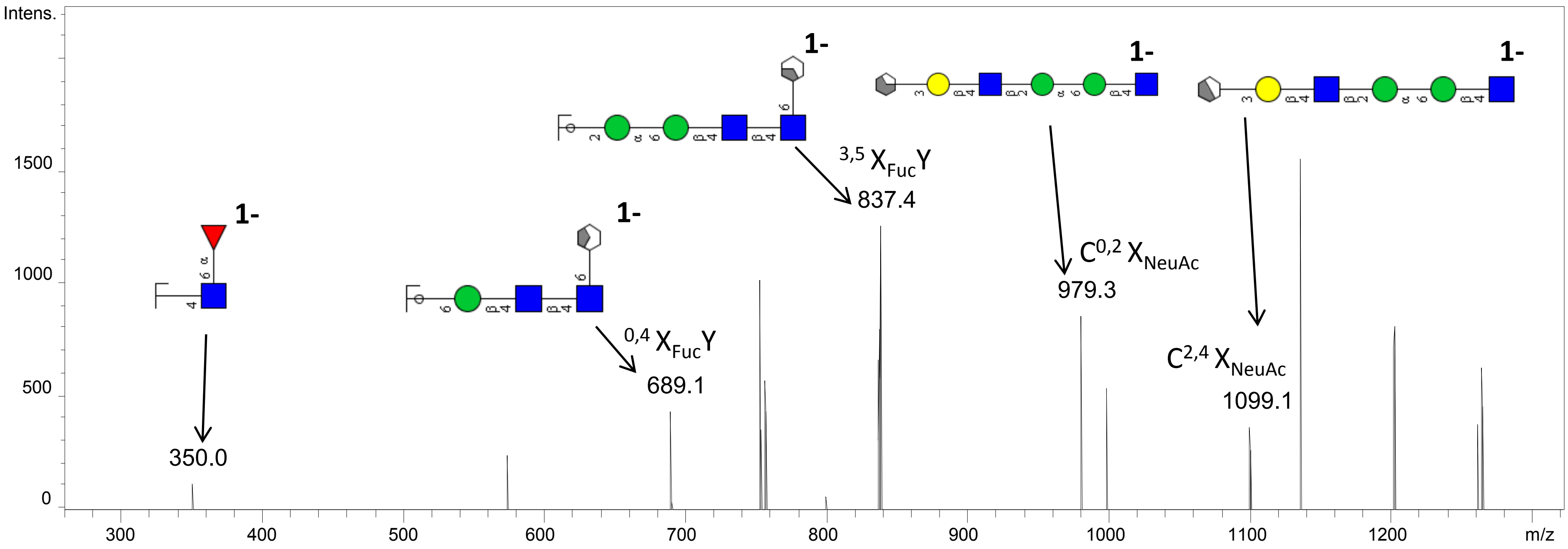

Glycan # 8

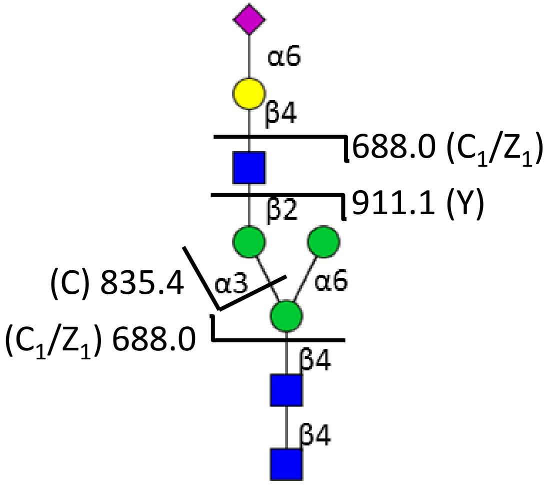

Observed *m/z* 783.3 (2-), RT: 42.2 min
$[M\text{-}H]^-$ 1567.6

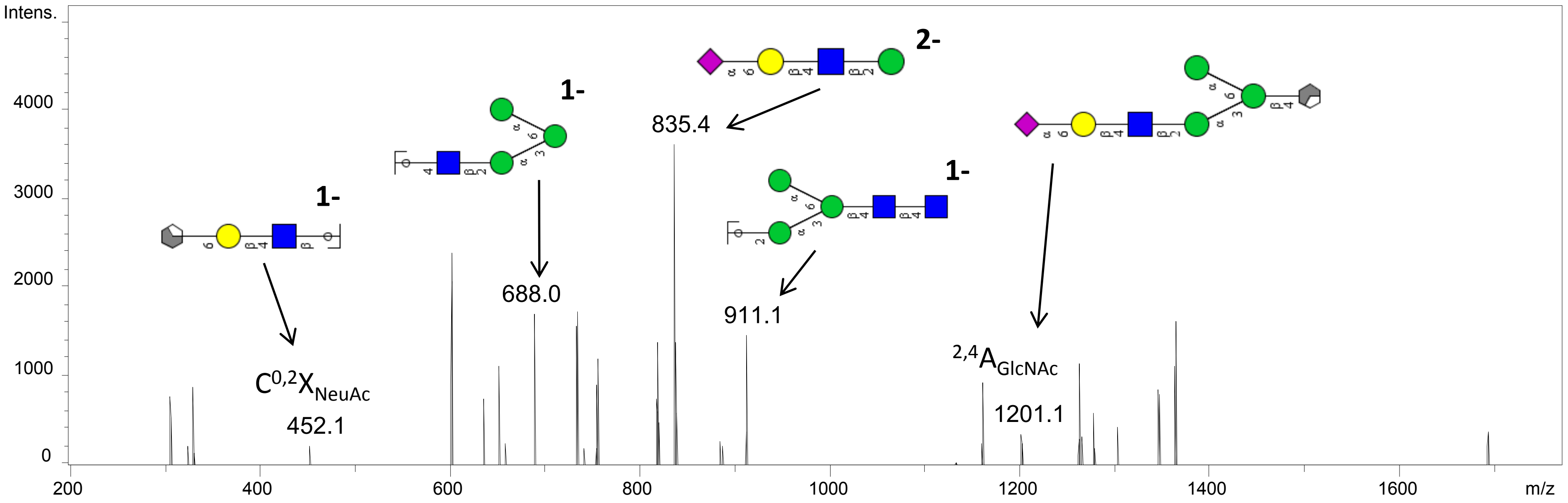

Glycan # 9a

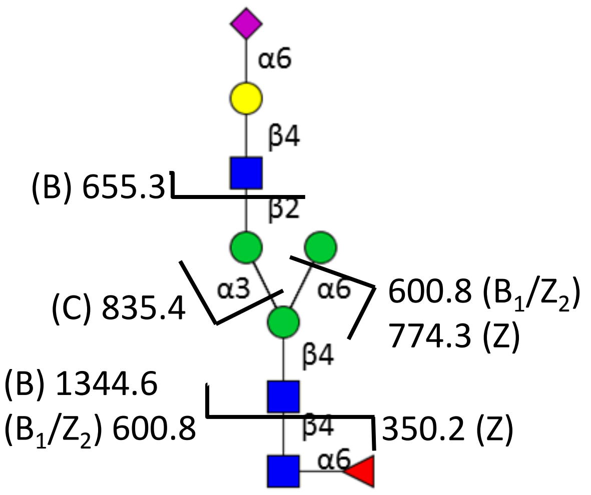

Observed *m/z* 856.4 (2-), RT: 47.5 min

[M-H]⁻ 1713.8

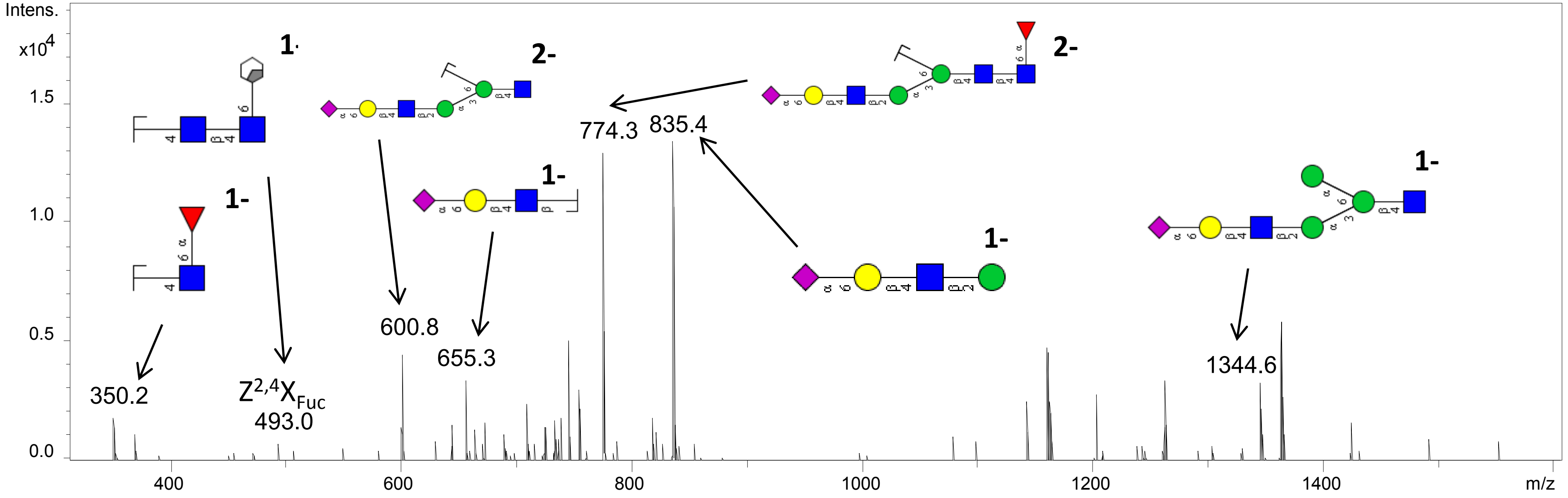

Glycan # 9b

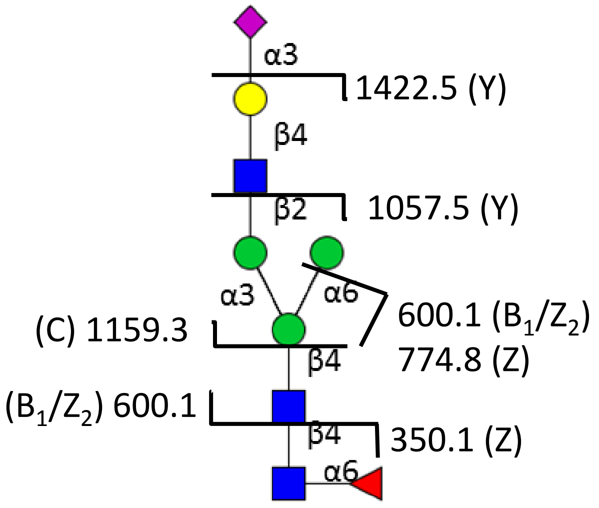

Observed *m/z* 856.4 (2-), RT: 55.1 min

$[M-H]^-$ 1713.8

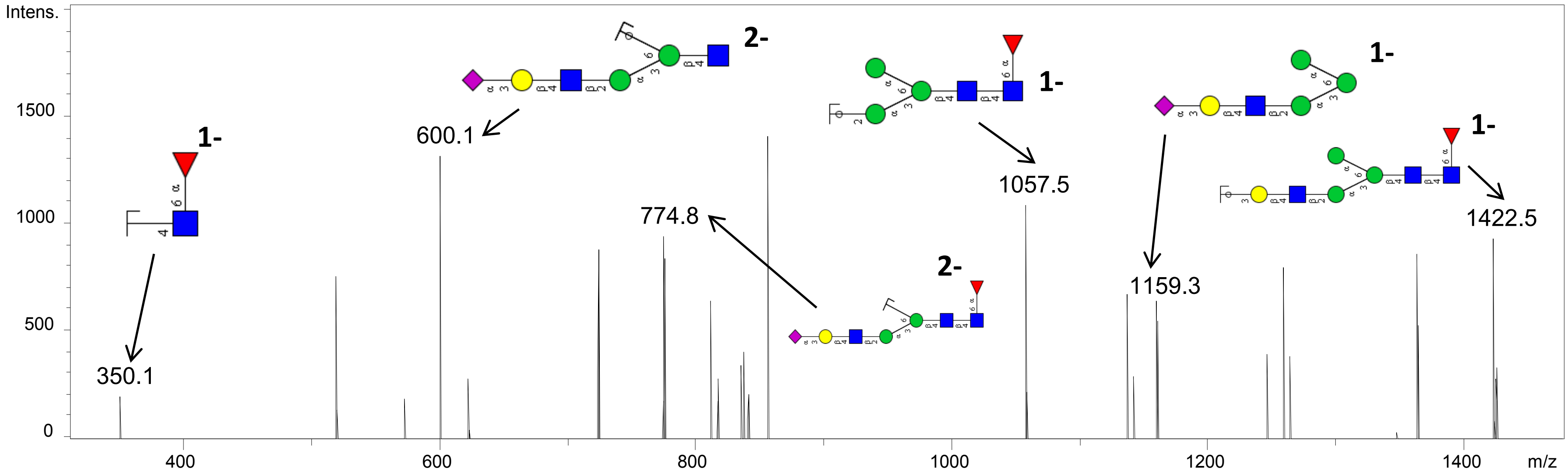

Supplementary Figure S4. Annotated RP-LC-ESI-CID/ETD-MS/MS spectra of all *N*-glycopeptides identified from the unenriched chymotryptic peptide mixture of the nCG protein preparation. CID-MS/MS spectra of the nCG C-terminal variants i.e. Arg243 and Ser244 C-terminal peptides are also shown.

Human neutrophil cathepsin G – P08311

IRTTMR (Arg243)

Obs. *m/z* 389.3 (2+)

Obs. $[M+H]^+$ = 777.6 Da

Arginine terminating C-terminal variant

Calc. $[M+H]^+$ = 777.4 Da

Retention time: 18.2 min

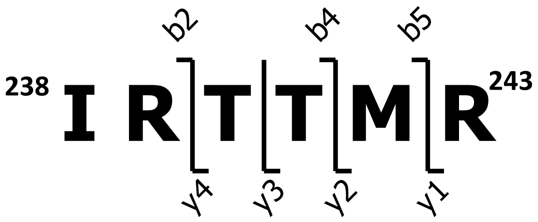

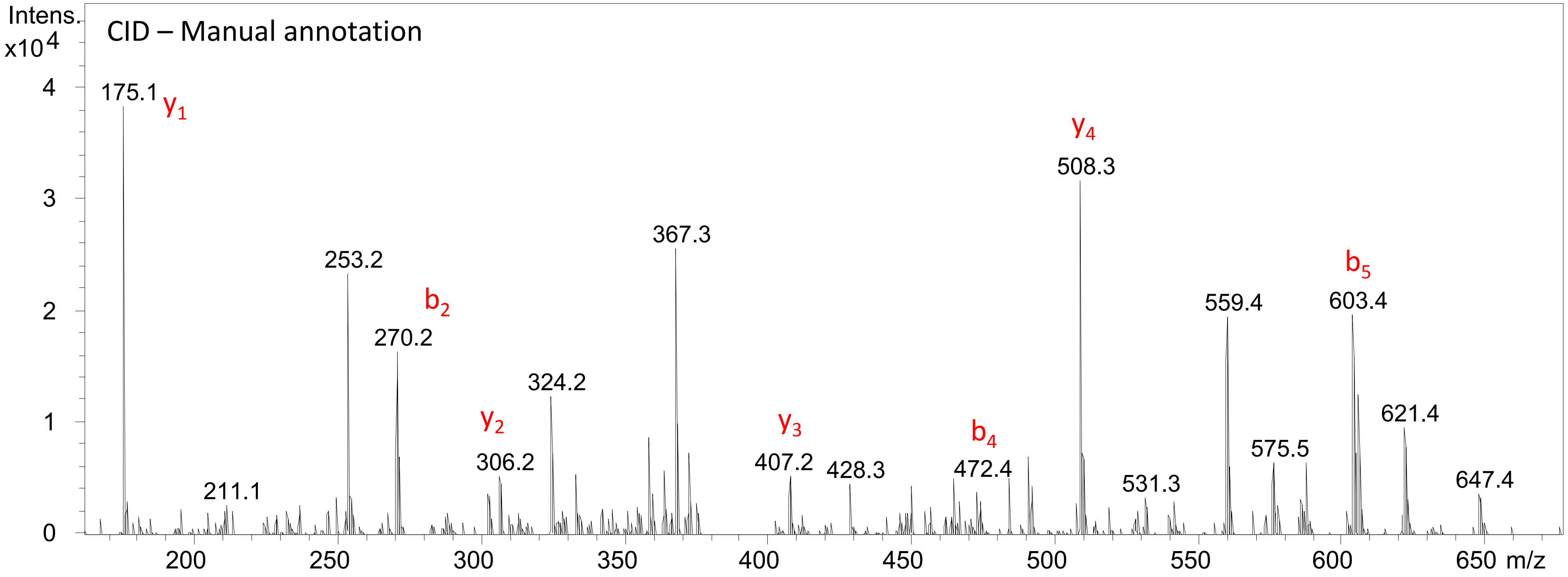

Human neutrophil cathepsin G – P08311

IRTTMRS (Ser244)

Obs. *m/z* 432.9 (2+)

Obs. $[M+H]^+$ = 864.8 Da

Serine terminating C-terminal variant

Calc. $[M+H]^+$ = 864.4 Da

Retention time: 18.3 min

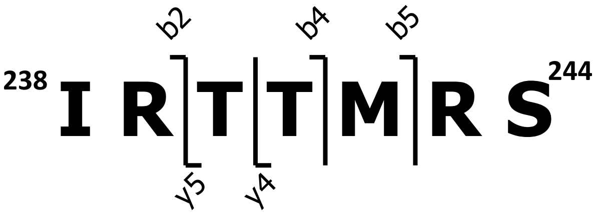

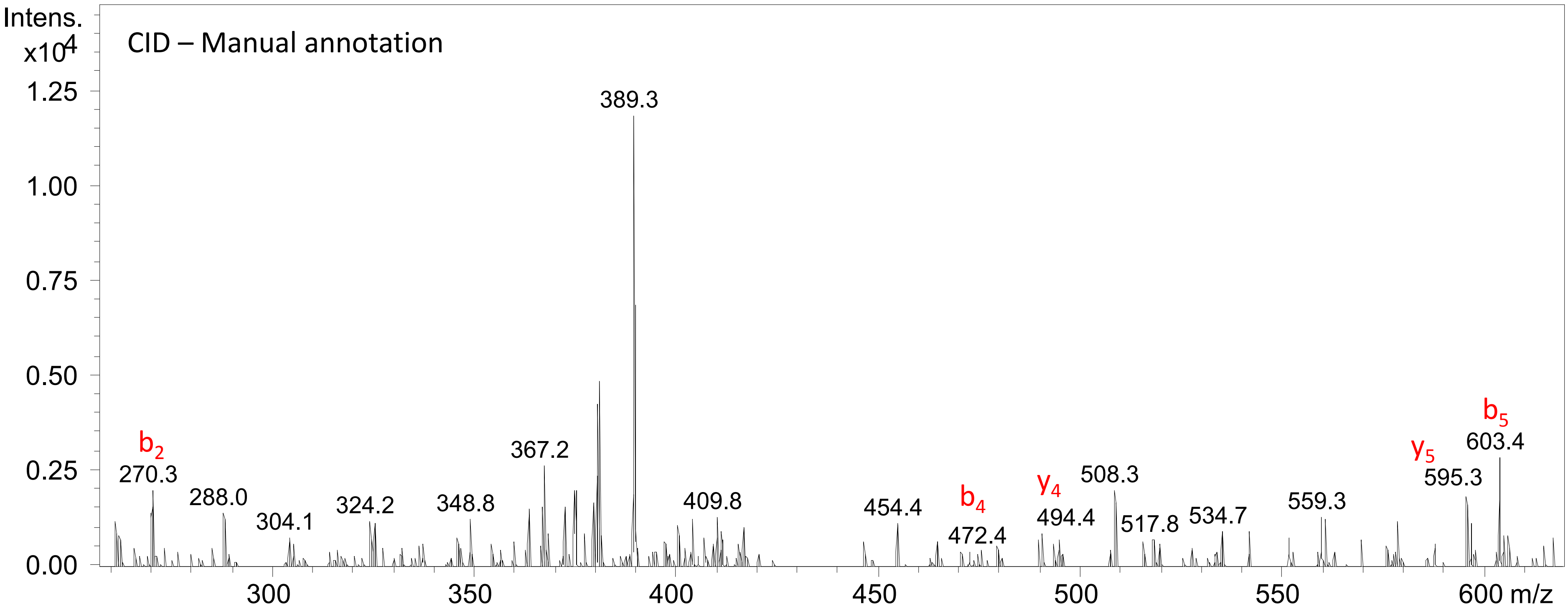

Human neutrophil cathepsin G – P08311

GSNI**N**VTL (Asn71)

Obs. *m/z* 510.8 (2+)

Obs. $[M+H]^+$ = 1020.6 Da

Glycan: 204.09 Da ($HexNAc_1$)

Calc. $[M+H]^+$ = 1020.5 Da

Retention time: 39.7 min

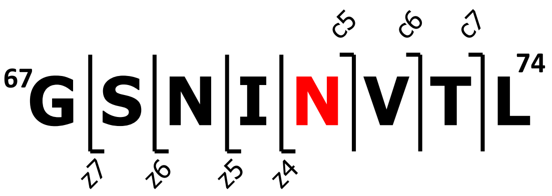

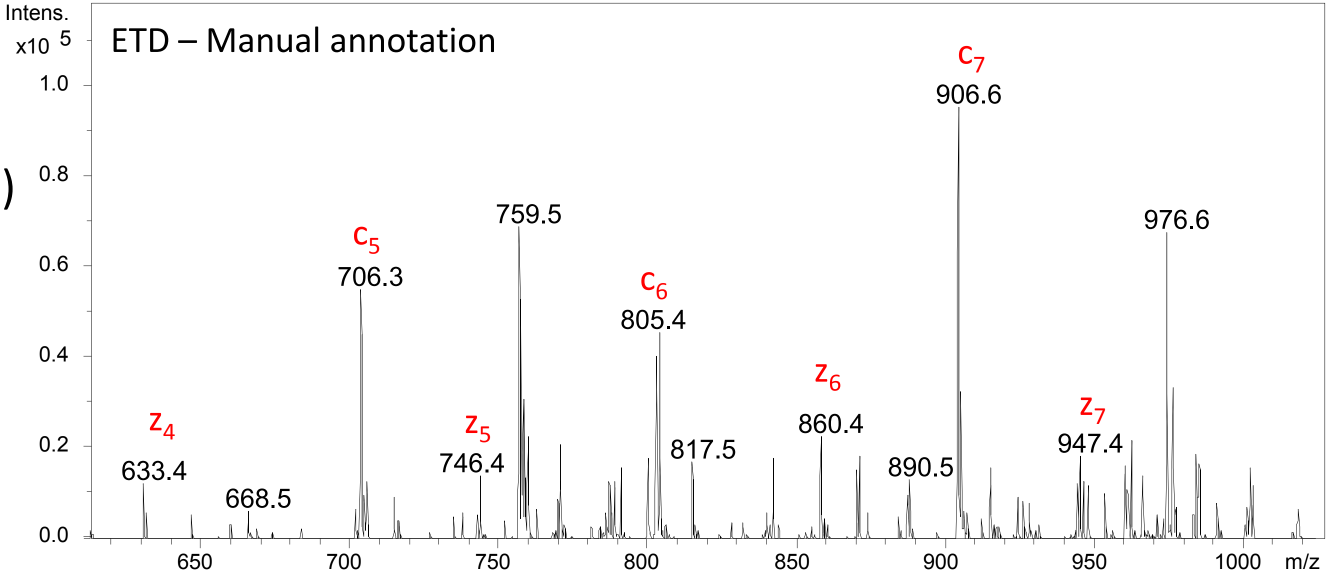

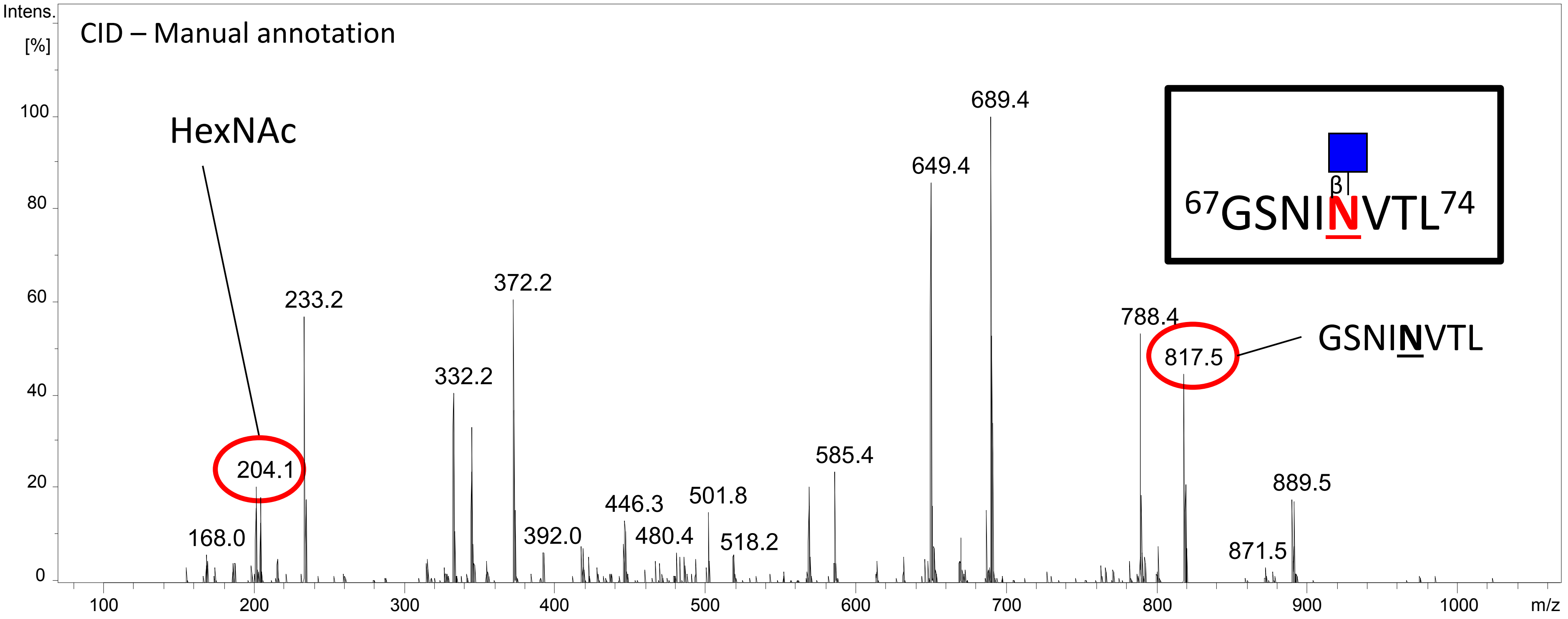

Human neutrophil cathepsin G – P08311

GSNI**N**VTL (Asn71)

Obs. *m/z* 583.8 (2+)

Obs. $[M+H]^+$ = 1166.6 Da

Glycan: 349.1 Da ($Fuc_1HexNAc_1$)

Calc. $[M+H]^+$ = 1166.5 Da

Retention time: 39.3 min

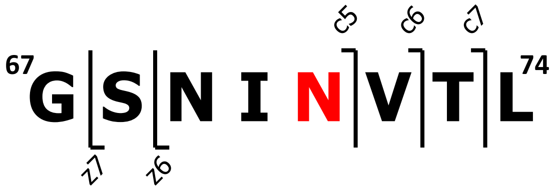

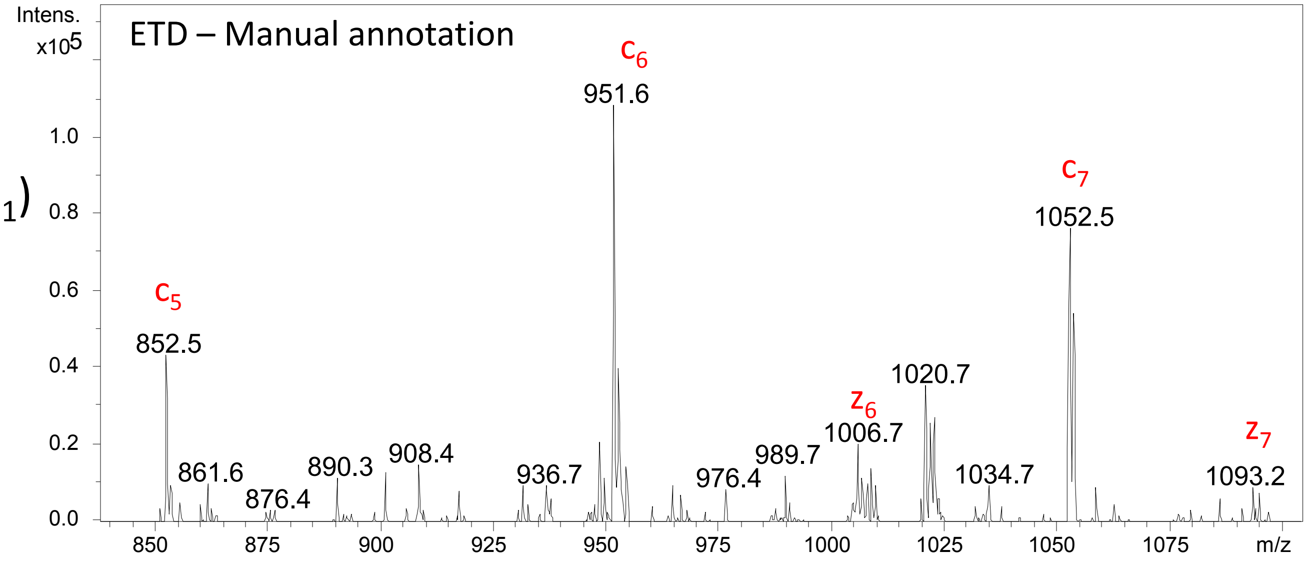

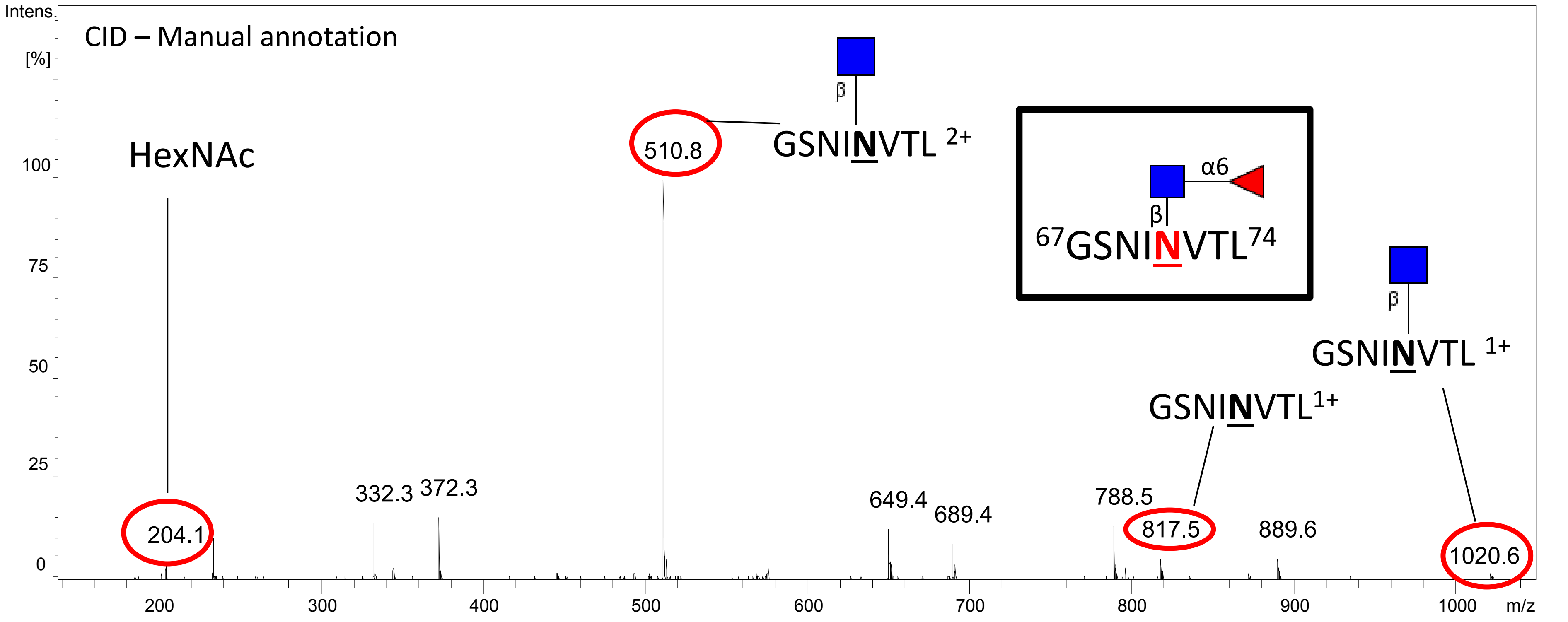

Human neutrophil cathepsin G – P08311

GSNI**N**VTL (Asn71)

Obs. *m/z* 693.8 (2+)

Obs. $[M+H]^+$ = 1386.6 Da

Glycan: 568.2 Da ($Hex_1HexNAc_2$)

Calc. $[M+H]^+$ = 1385.6 Da

Retention time: 39.6 min

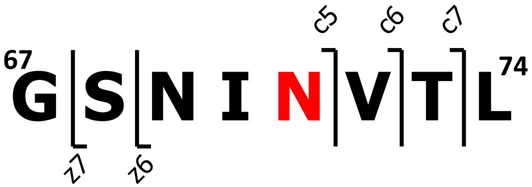

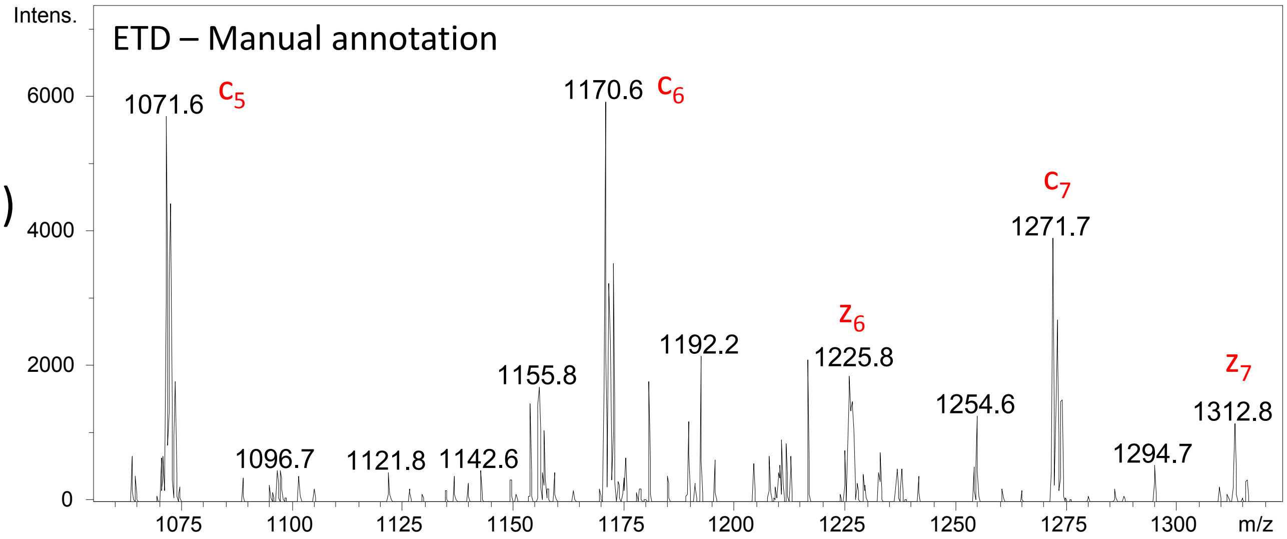

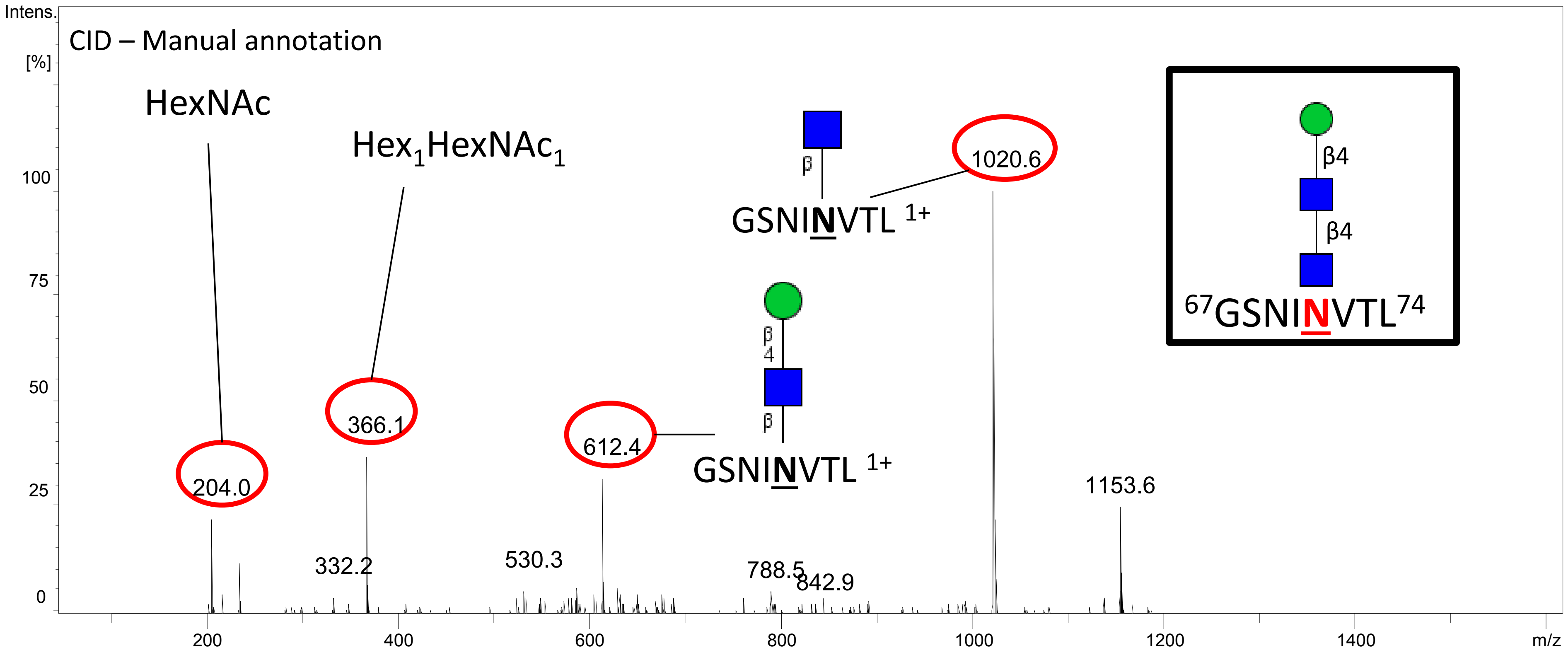

Human neutrophil cathepsin G – P08311

GSNI**N**V (Asn71)

Obs. *m/z* 586.7 (2+)

Obs. $[M+H]^+$ = 1172.4 Da

Glycan: 568.2 Da ($Hex_1HexNAc_2$)

Calc. $[M+H]^+$ = 1171.5 Da

Retention time: 24.7 min

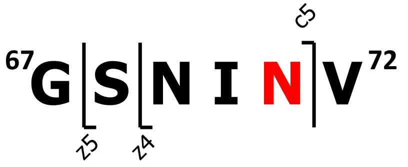

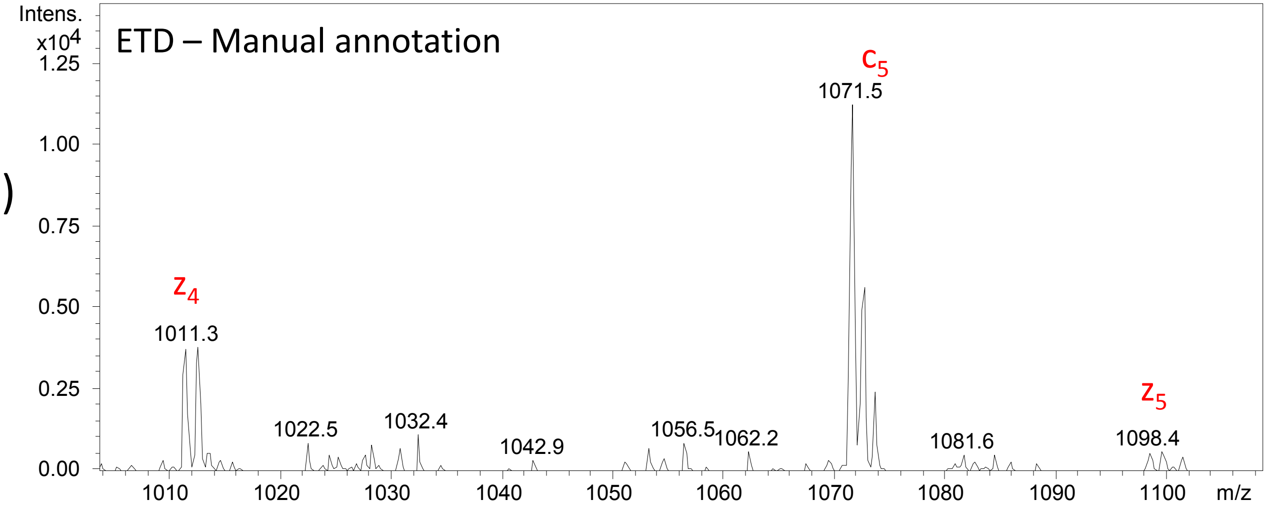

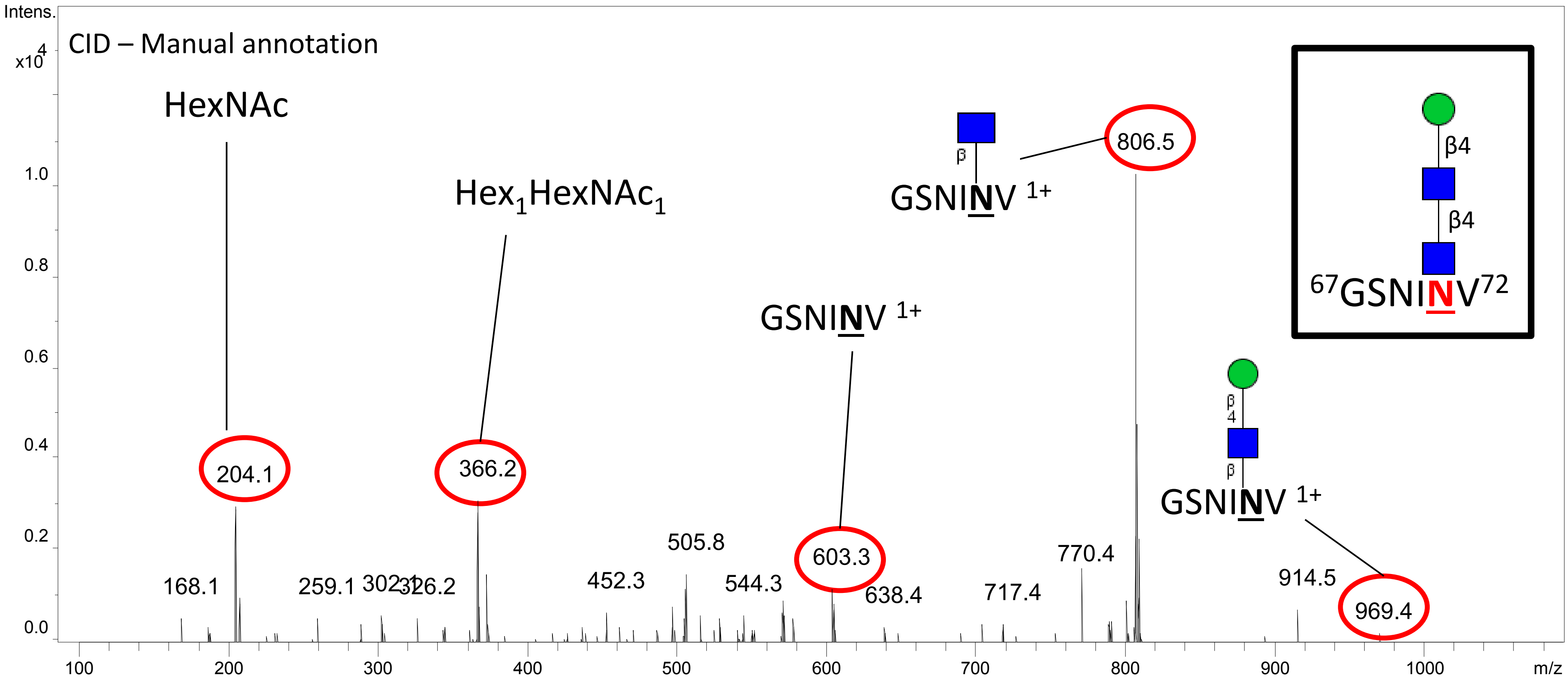

Human neutrophil cathepsin G – P08311

GSNI**N**V (Asn71)

Obs. *m/z* 659.4 (2+)

Obs. $[M+H]^+$ = 1317.8 Da

Glycan: 714.2 Da ($Fuc_1Hex_1HexNAc_2$)

Calc. $[M+H]^+$ = 1317.5 Da

Retention time: 26.1 min

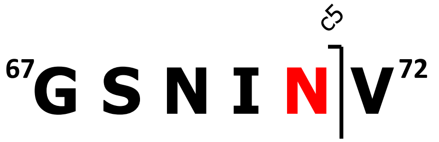

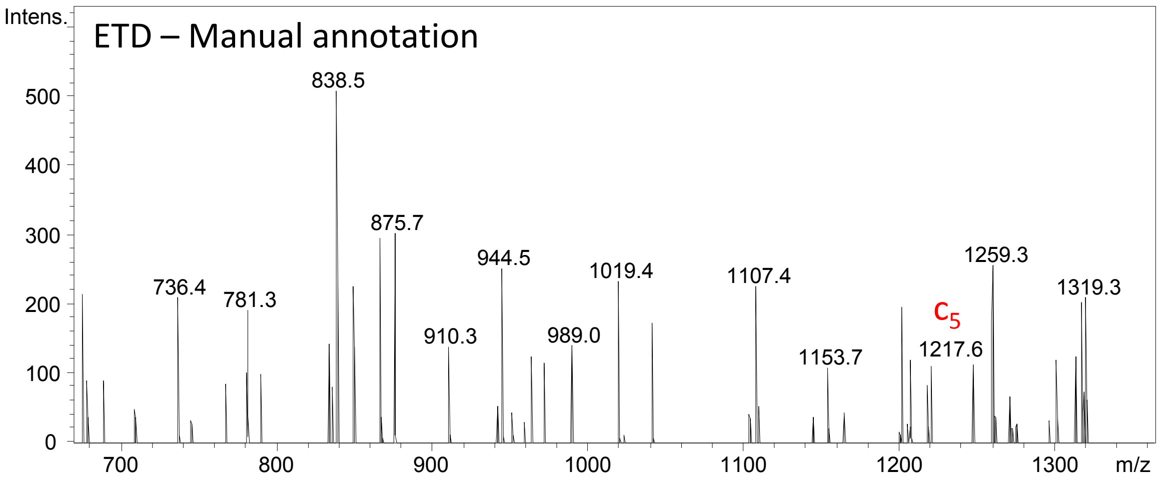

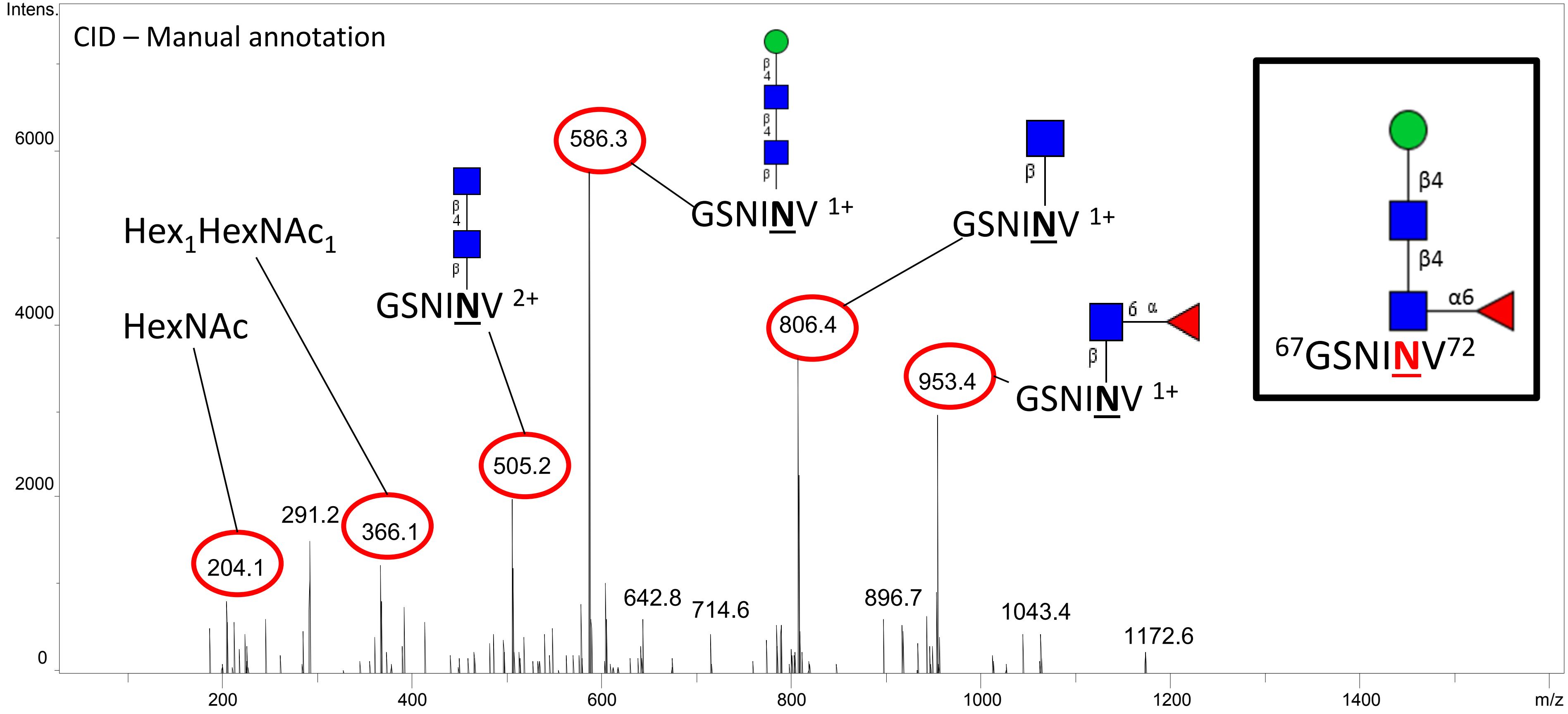

Human neutrophil cathepsin G – P08311

GSNI**N**V (Asn71)

Obs. *m/z* 667.8 (2+)

Obs. $[M+H]^+$ = 1334.6 Da

Glycan: 730.2 Da ($Hex_2HexNAc_2$)

Calc. $[M+H]^+$ = 1333.5 Da

Retention time: 24.4 min

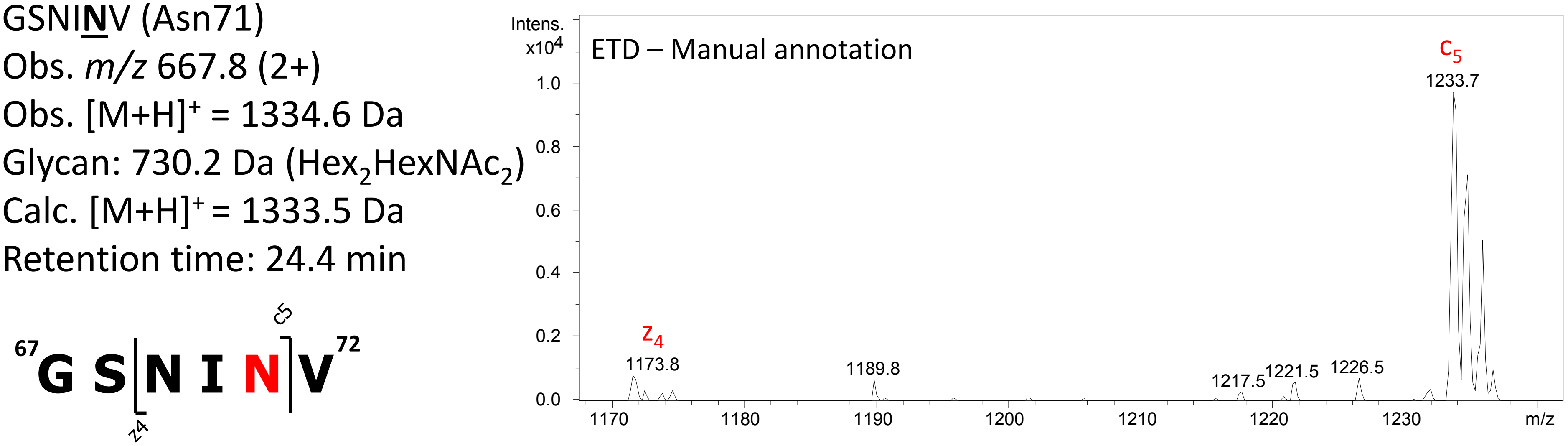

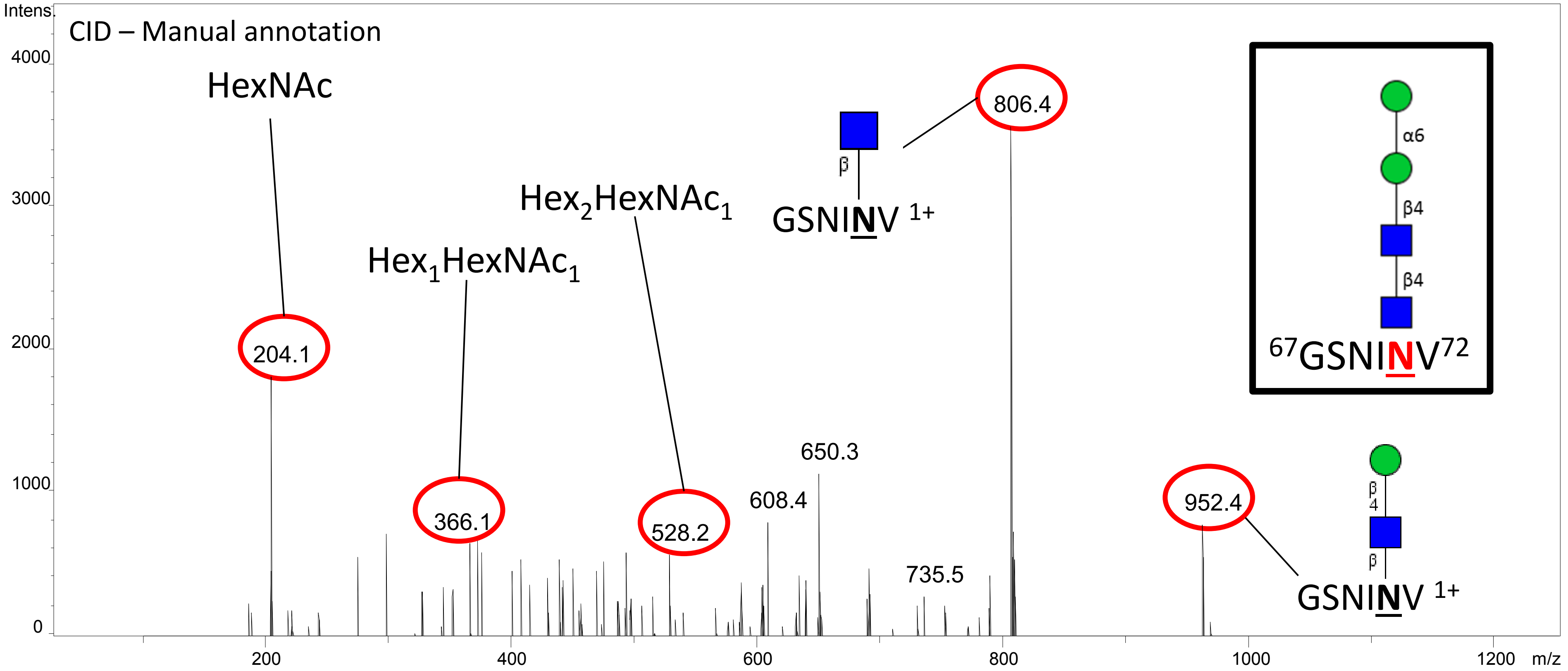

Human azurocidin – P20160

SRFPRFV**N**V (Asn171)

Obs. *m/z* 612.7 (3+)

Obs. $[M+H]^+$ = 1836.1 Da

Glycan: 714.2 Da ($Fuc_1Hex_1HexNAc_2$)

Calc. $[M+H]^+$ = 1835.8 Da

Retention time: 43.0 min

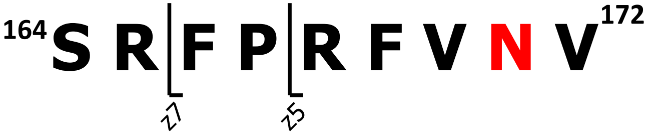

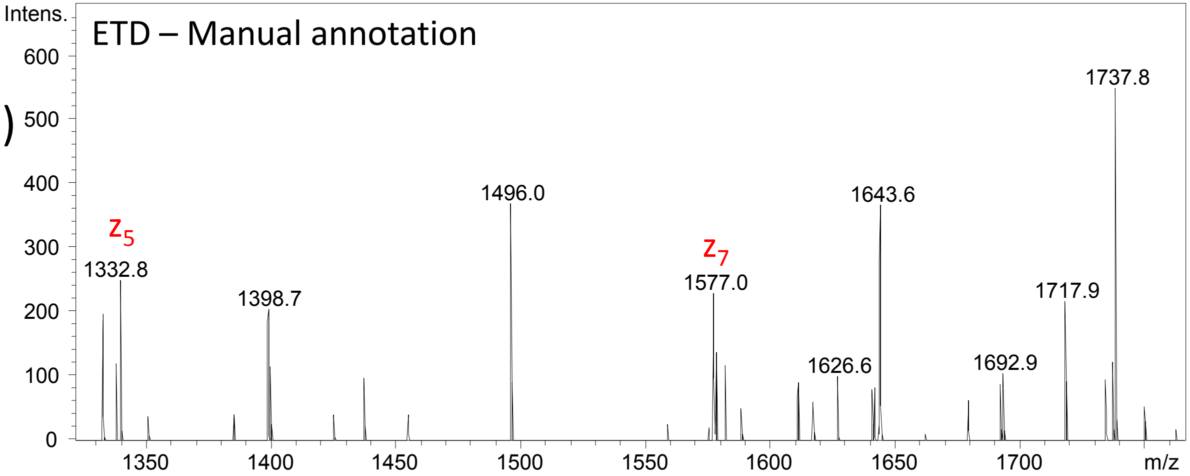

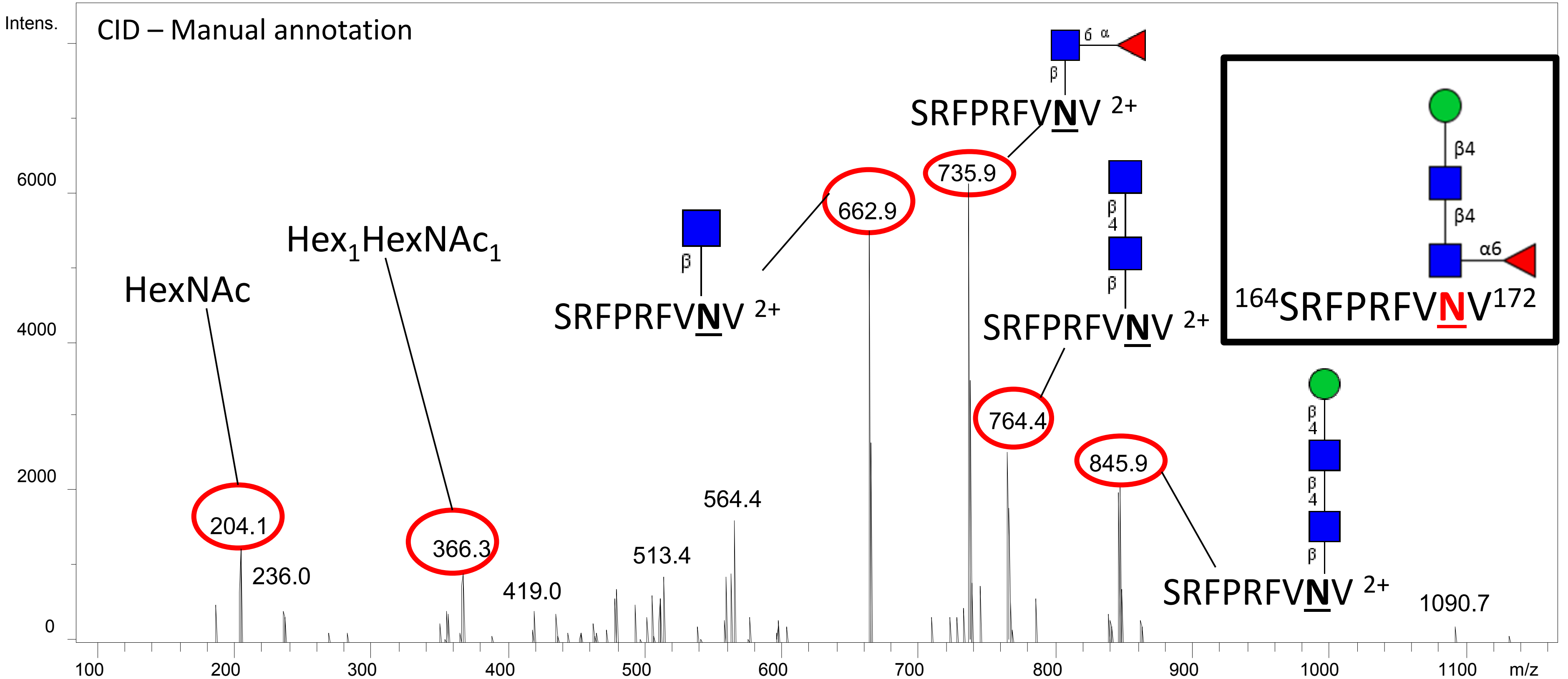

Human azurocidin – P20160

REA**N**L (Asn126)

Obs. *m/z* 739.5 (2+)

Obs. $[M+H]^+$ = 1478.6 Da

Glycan: 876.8 Da ($Fuc_1Hex_2HexNAc_2$)

Calc. $[M+H]^+$ = 1478.8 Da

Retention time: 19.1 min

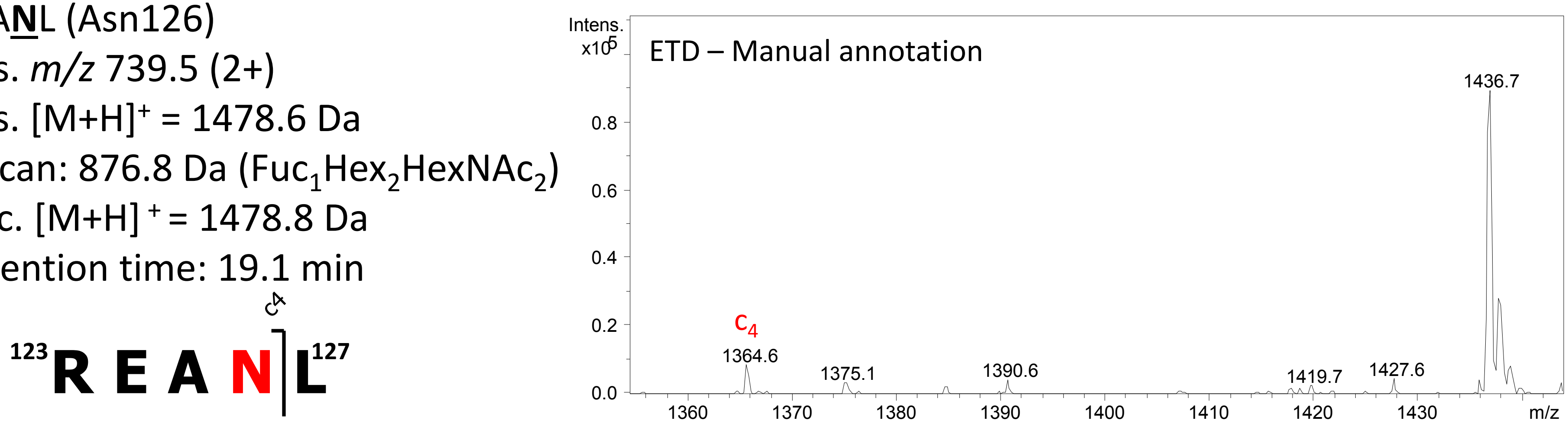

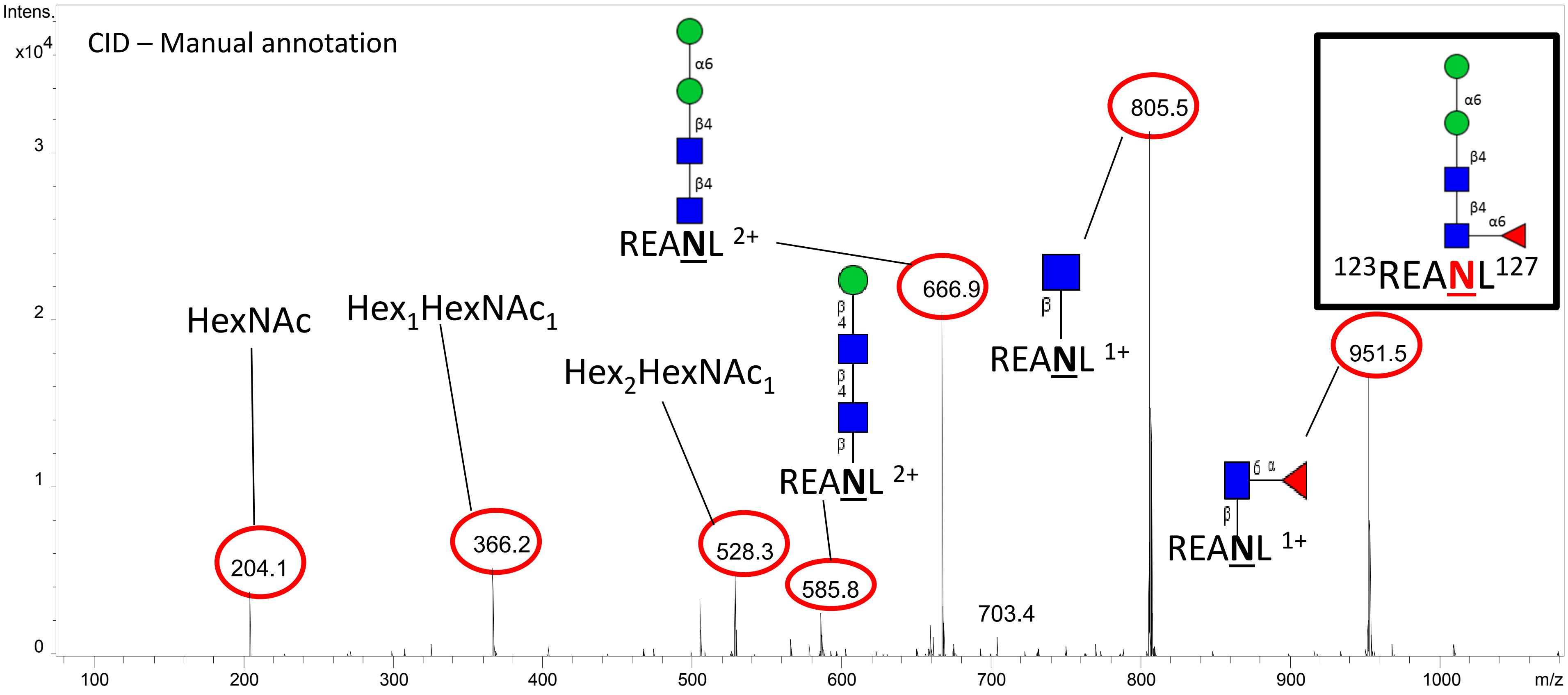

Human azurocidin – P20160

REA**N**LTSSV (Asn126)

Obs. *m/z* 927.0 (2+)

Obs. $[M+H]^+$ = 1853.0 Da

Glycan: 876.8 Da ($Fuc_1Hex_2HexNAc_2$)

Calc. $[M+H]^+$ = 1852.8 Da

Retention time: 25.8 min

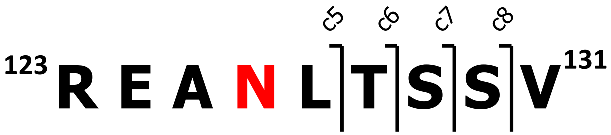

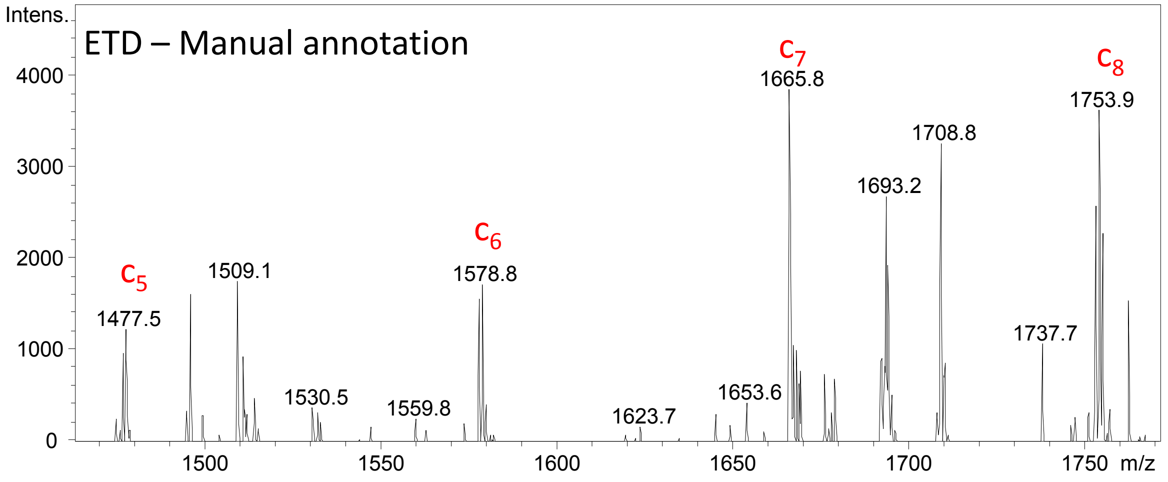

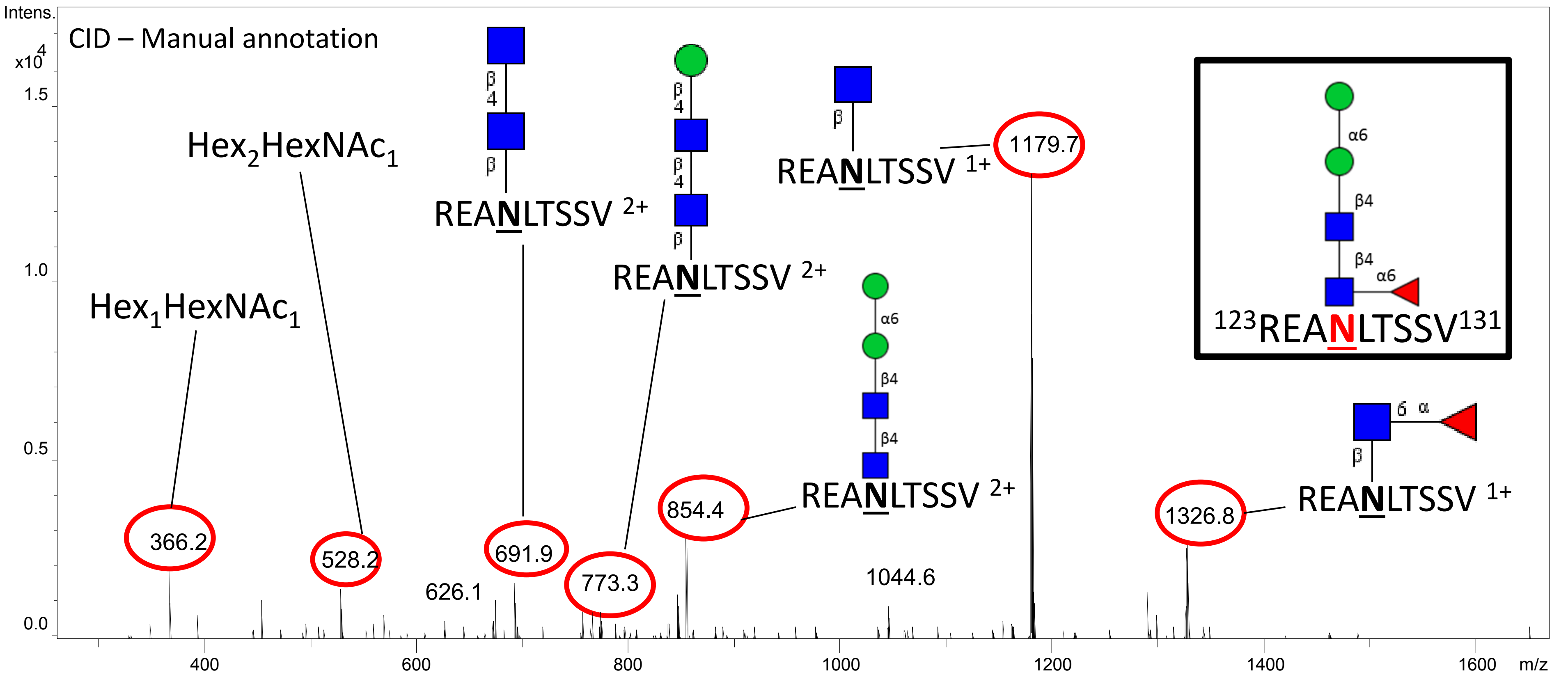

Human azurocidin – P20160

PRFV**N**V (Asn171)

Obs. *m/z* 804.9 (2+)

Obs. $[M+H]^+$ = 1608.8 Da

Glycan: 876.8 Da ($Fuc_1Hex_2HexNAc_2$)

Calc. $[M+H]^+$ = 1607.8 Da

Retention time: 32.6 min

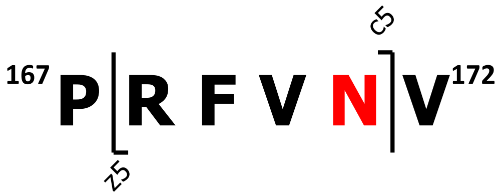

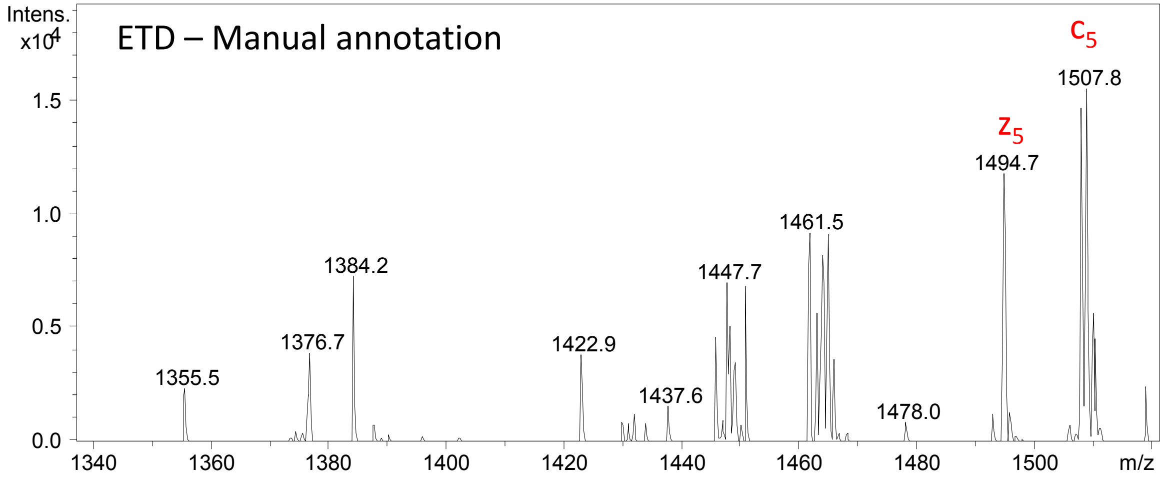

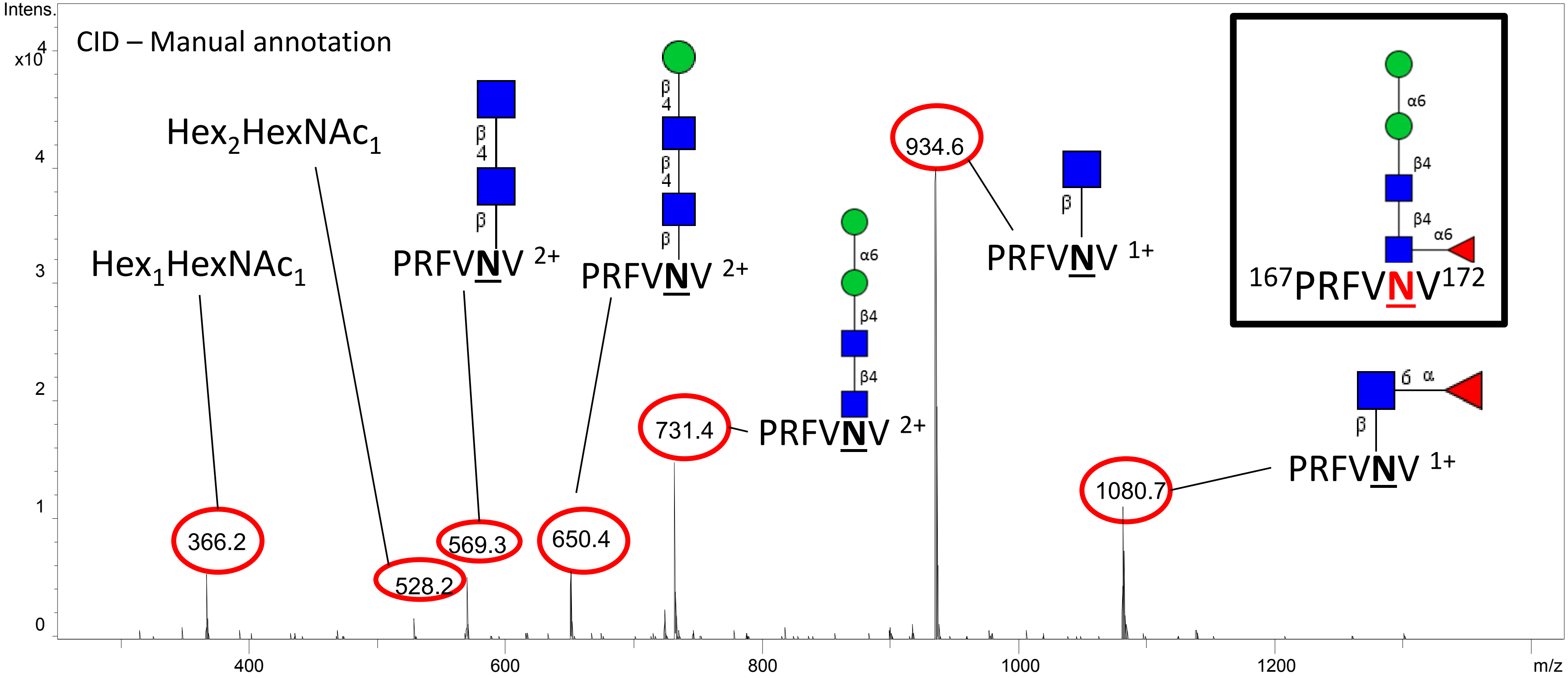

Human neutrophil elastase – P08246

QEL**N**VTVV (Asn173)

Obs. *m/z* 889.4 (2+)

Obs. $[M+H]^+$ = 1777.8 Da

Glycan: 876.8 Da ($Fuc_1Hex_2HexNAc_2$)

Calc. $[M+H]^+$ = 1777.8 Da

Retention time: 40.4 min

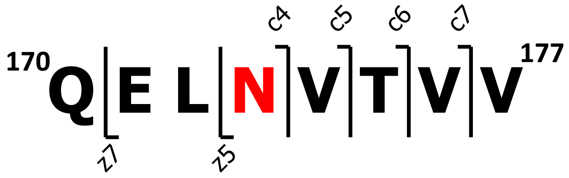

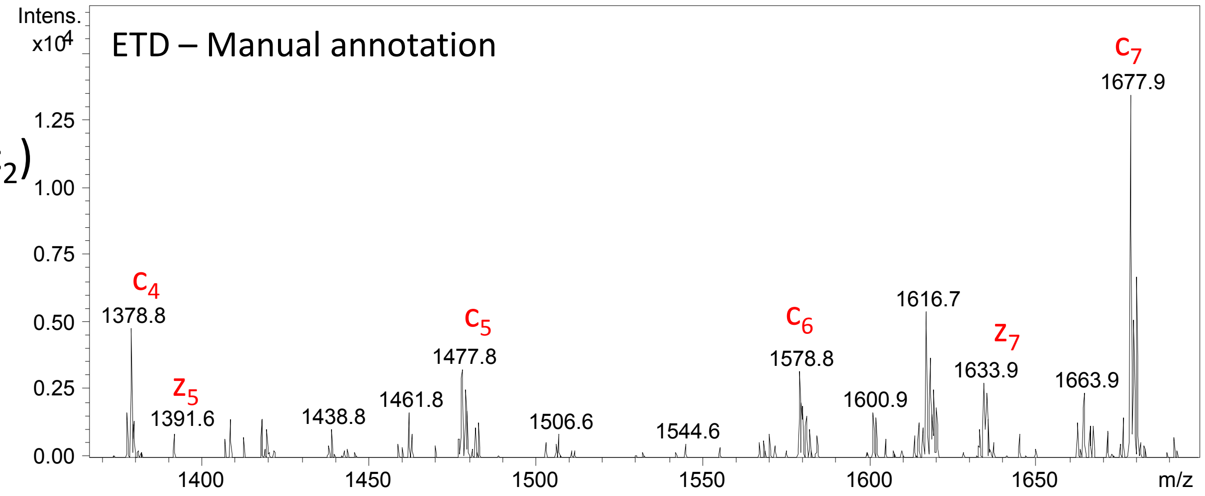

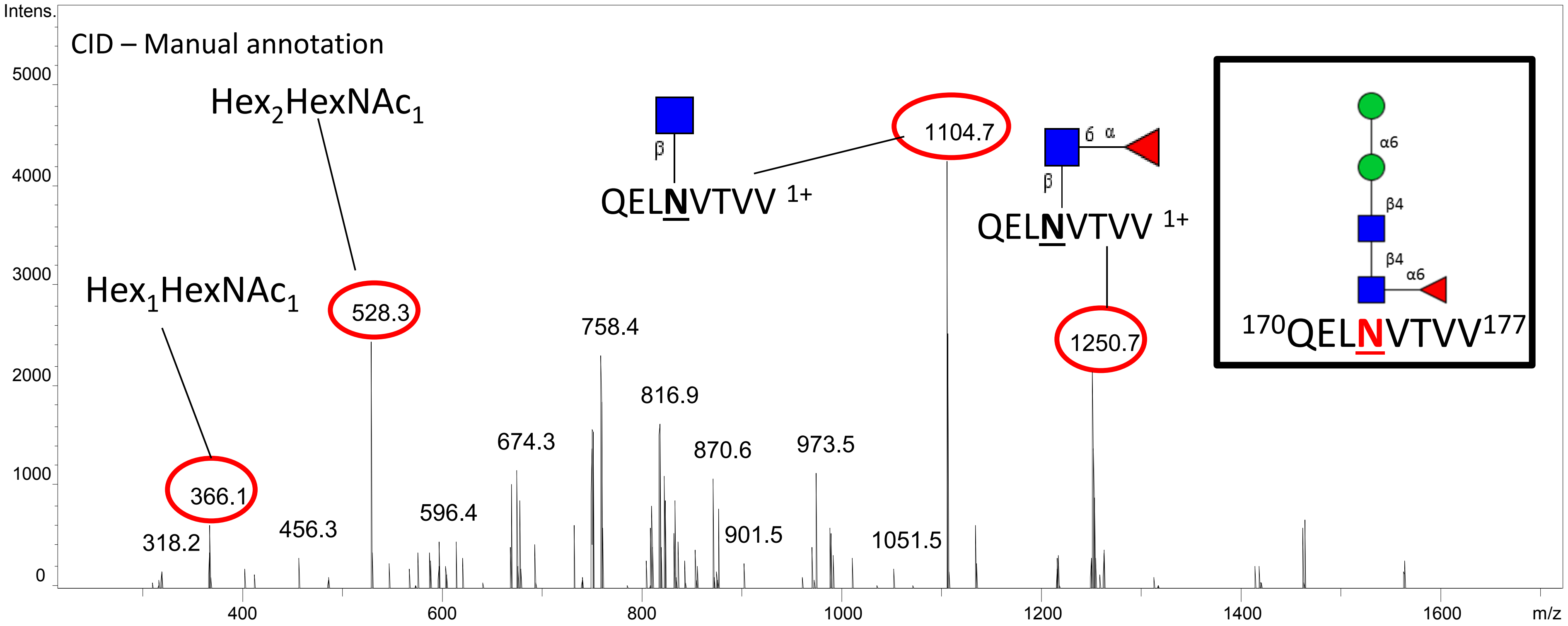

Human neutrophil elastase – P08246

QL<u>N</u>GSATI (Asn124)

Obs. *m/z* 840.0 (2+)

Obs. $[M+H]^+$ = 1679.0 Da

Glycan: 876.8 Da ($Fuc_1Hex_2HexNAc_2$)

Calc. $[M+H]^+$ = 1679.7 Da

Retention time: 28.0 min

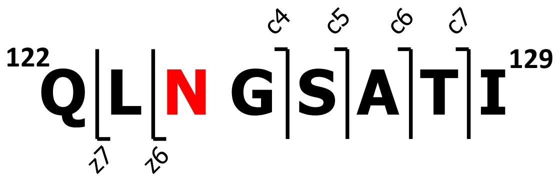

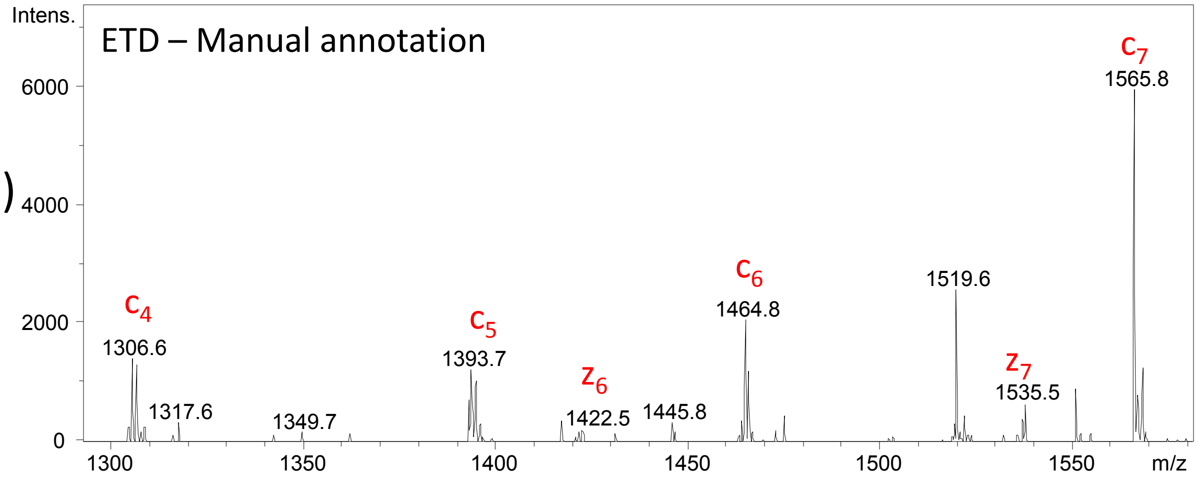

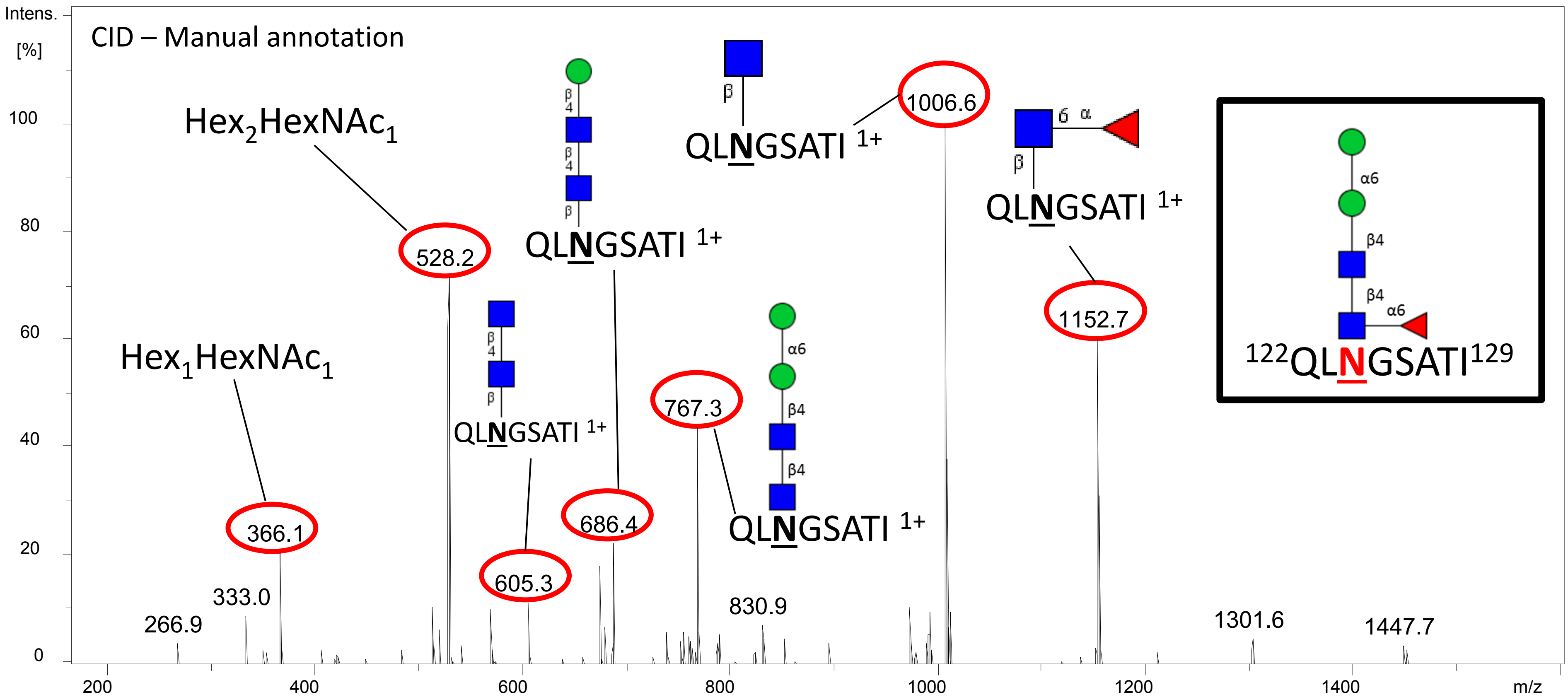

Supplementary Figure S5. Presence of oxidised (top) and absence of non-oxidised (bottom) Met152 on a chymotryptic peptide of nCG as evaluated using RP-LC-ESI-ETD-MS/MS.
* Monoisotopic precursor.

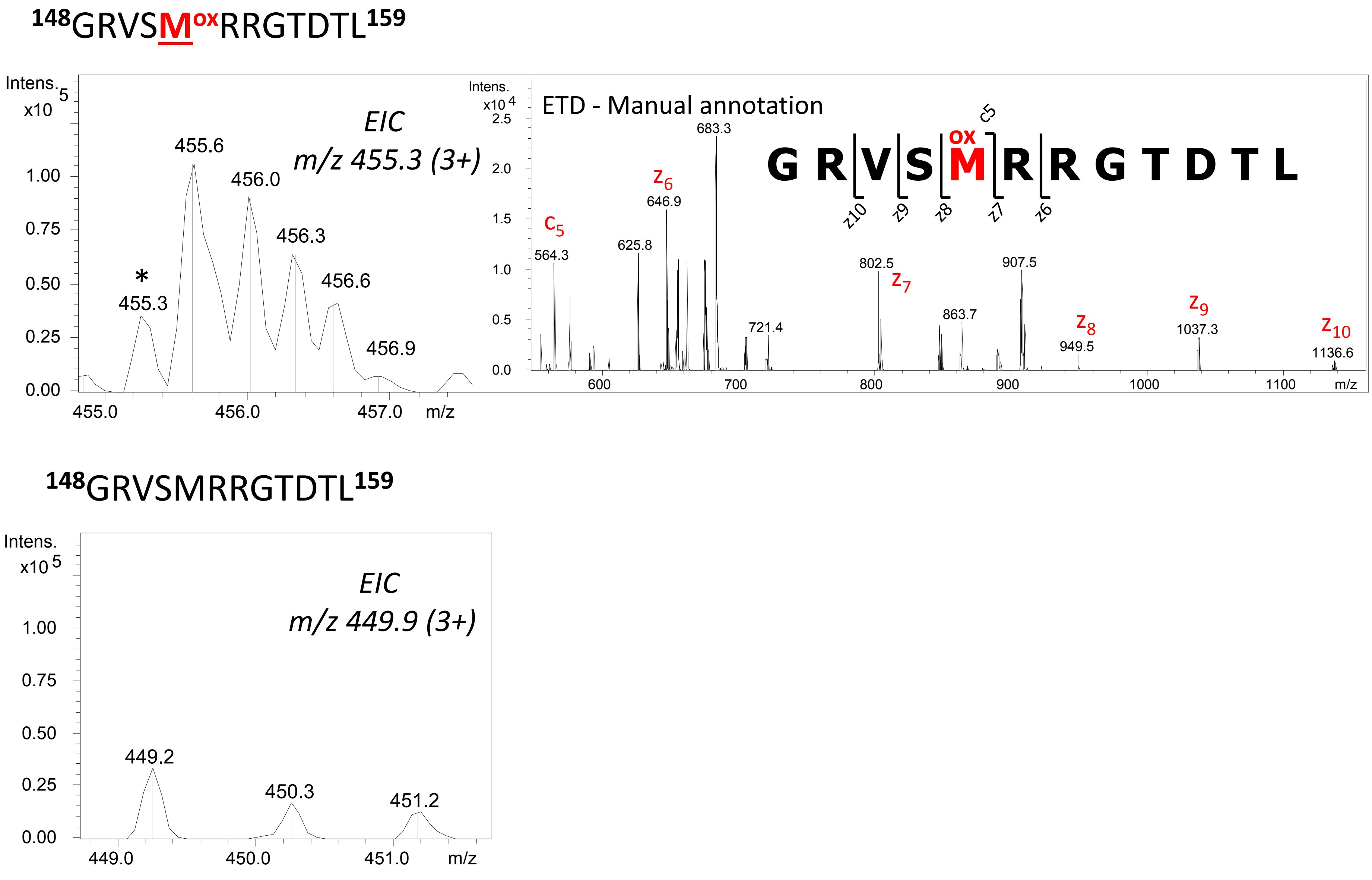

Supplementary Figure S6. Comparison of high and low fragmentor potential when profiling intact nCG using high resolution QTOF-ESI-MS. The masses after deconvolution of nCG Arg243 and Ser244 C-terminal variants are presented in black and red, respectively. -ox denotes Met152 oxidation. § denotes water loss. * denotes adduct formation.

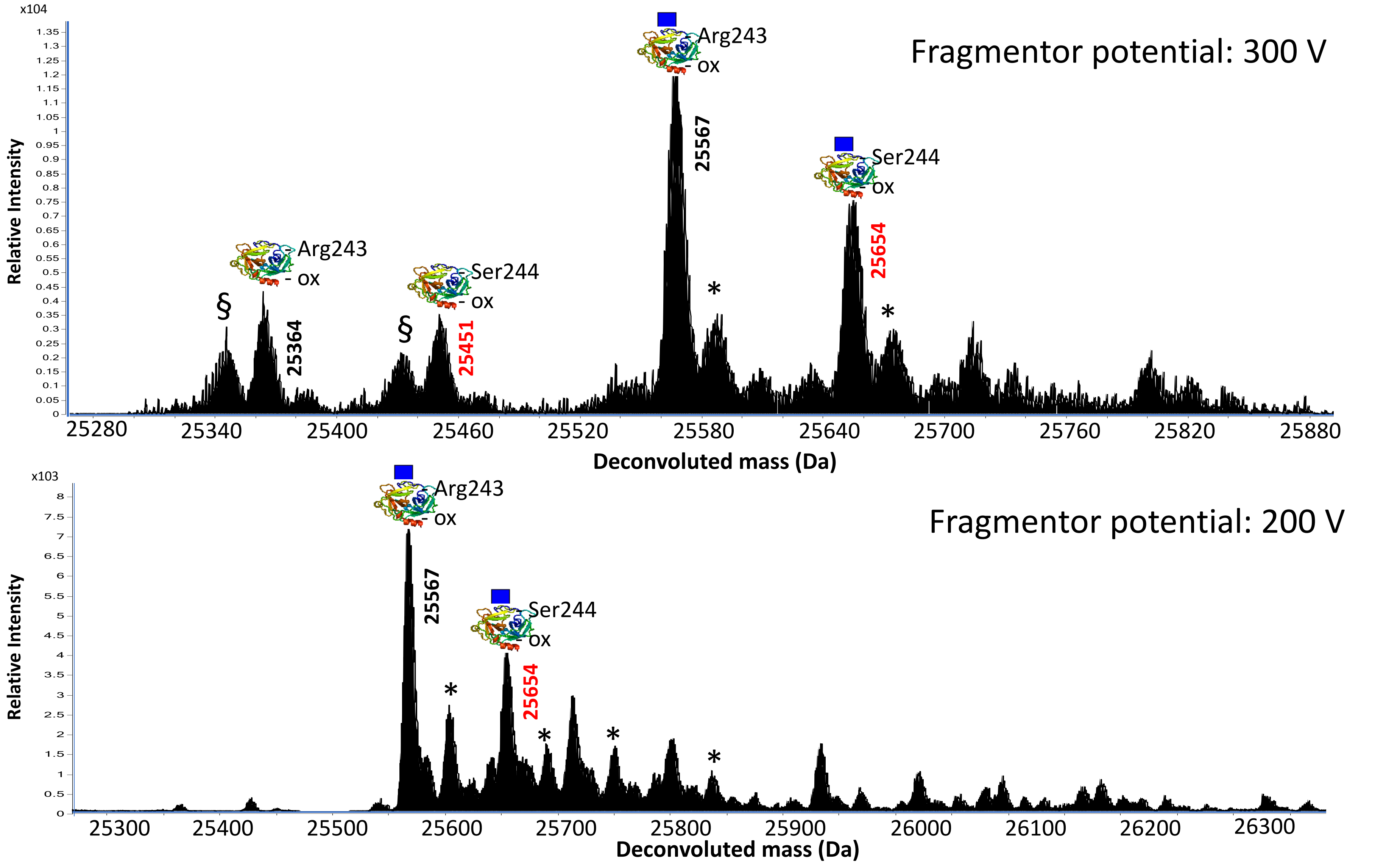

Supplementary Figure S7. *N*-glycosidase F-resistance of truncated chitobiose core Asn71-glycopeptides of nCG (bottom MS) but not of others e.g. M1 N-glycans (top BPC and EIC).

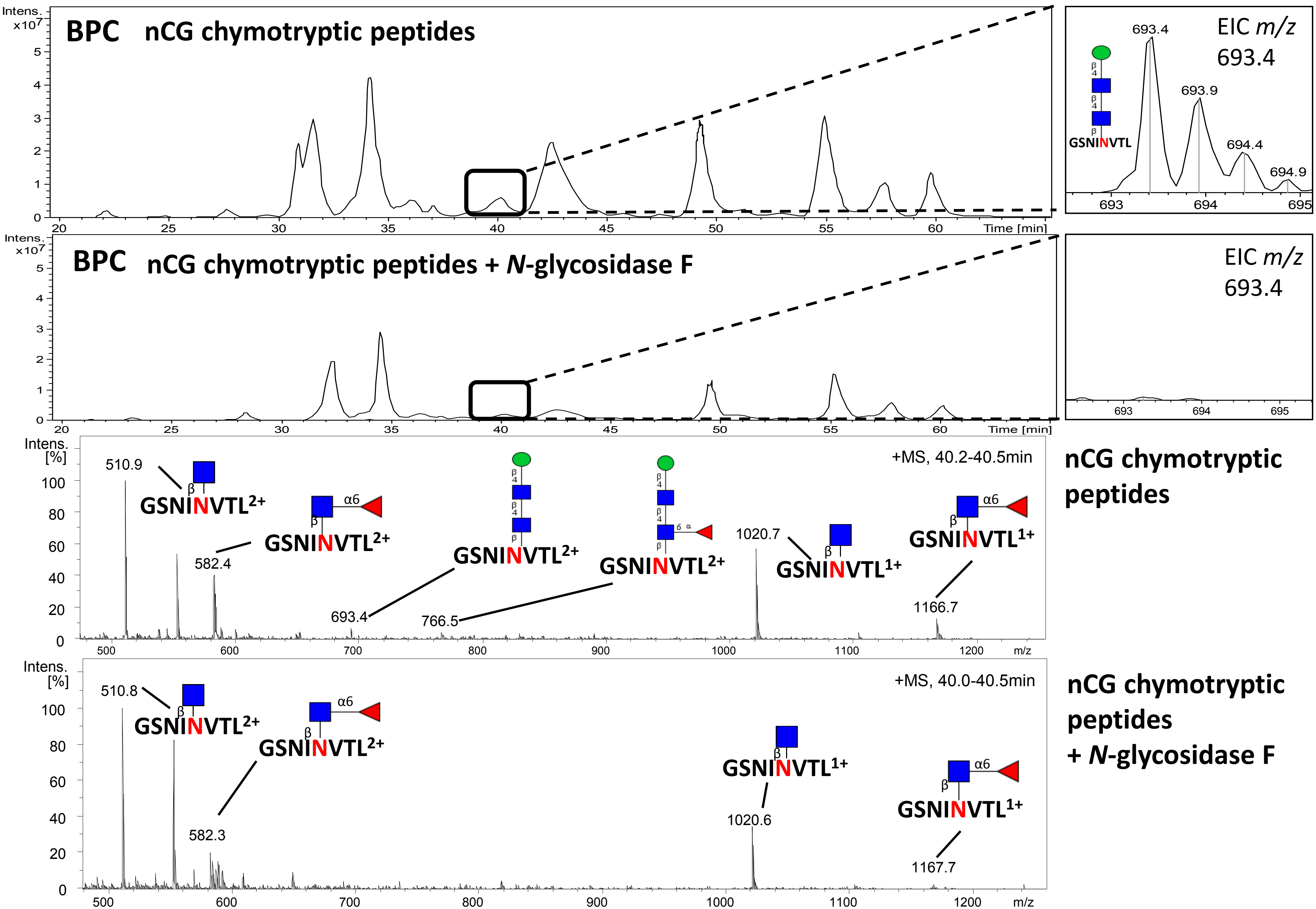

Supplementary Figure S8. 3D modelling of the trimannosyl-chitobiose core monoantennary core fucosylated α2,6-monosialylated *N*-glycan on Asn71 of nCG. The height of the *N*-glycan from the asparagine base (~27 Å) is longer than the distance to the active site (~19 Å).

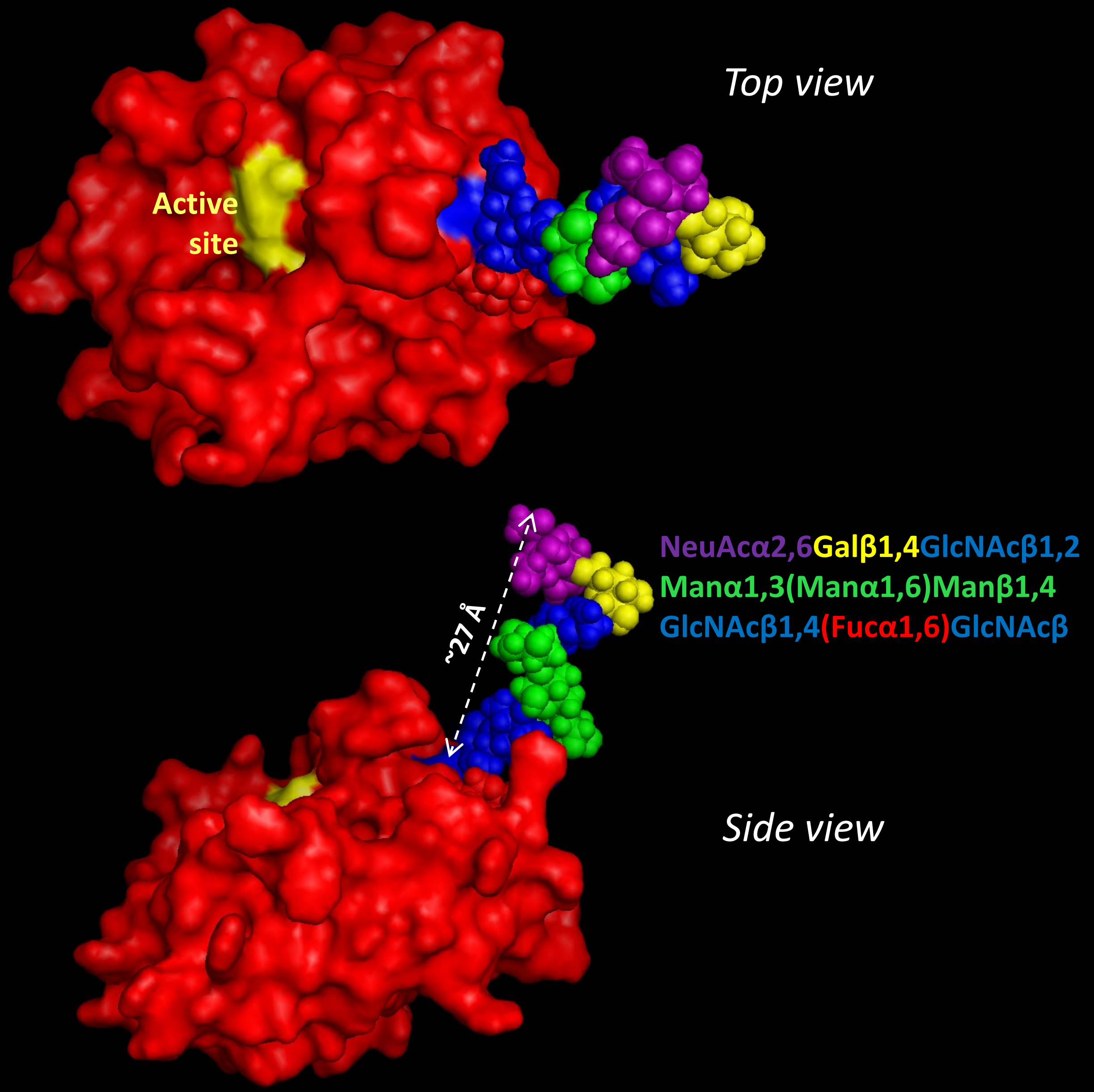

Supplement: Supplementary File 1 [file biomolecules-05-01832-s001.zip › biomolecules-89812-Supplementary Figures and Table S1-update.pdf]
